# A Project Based Learning Approach for Improving Students’ Computational Thinking Skills

**DOI:** 10.3389/frobt.2022.720448

**Published:** 2022-03-07

**Authors:** Francesca Bertacchini, Carmelo Scuro, Pietro Pantano, Eleonora Bilotta

**Affiliations:** ^1^ Department of Mechanics, Energy and Management Engineering, University of Calabria, Rende, Italy; ^2^ Laboratory of Cognitive Science and Modelling, Department of Physics, University of Calabria, Rende, Italy; ^3^ Department of Physics, University of Calabria, Rende, Italy

**Keywords:** educational robotics, social robots, motor-behavior, project-based learning, mathematica wolfram language

## Abstract

An educational robotics lab has been planned for undergraduate students in an Electronic Engineering degree, using the Project Based Learning (PBL) approach and the NAO robot. Students worked in a research context, with the aim of making the functions of the NAO robot as social and autonomous as possible, adopting in the design process the Wolfram Language (WL), from the Mathematica software. Interfacing the programming environment of the NAO with Mathematica, they solved in part the problem of autonomy of the NAO, thus realizing enhanced functions of autonomous movement, recognition of human faces and speech for improving the system social interaction. An external repository was created to streamline processes and stow data that the robot can easily access. Self-assessment processes demonstrated that the course provided students with useful skills to cope with real life problems. Cognitive aspects of programming by WL have also been collected in the students’ feedback.

## Introduction

Programming social robots is becoming increasingly important, thanks to advances in robotic technologies and programming languages that manage the behavior of such systems in a user-friendly way. Commercially available social robotic platforms are widely used for coding activities dedicated to elementary, high and middle school students and their application have positive and negative impact on learning (Pachidis et al., 2019). Among the various existing applications, the NAO Robot, due to its size and functions, can perform very complex tasks in real-world environments (Bertacchini et al., 2017).

Educational robotics ties in with the activities that are spreading around the world related to coding. The use of robots in all educational processes from elementary to advanced education, which already began in the 2000s (Gerkey and Matarić, 2004; Berns and Braum, 2005; Frangou and Papanikolaou, 2009; Alimisis et al., 2009; Adamo et al., 2010; Eguchi, 2012), is a rapidly expanding research field. Students learn through building and programming small robots and social robots (Bers et al., 2014; Chen et al., 2017; Leonard et al., 2016). From some review articles (Mesquita et al., 2020; Johal et al., 2020), it is noted that robotics in education can be divided into three basic categories - robotics as a learning goal (Ahn et al., 2020), a learning aid (Miskon, et al., 2020), and a learning tool (Pozzi, et al., 2021). Robotics as a disciplinary goal focuses on the acquisition of technical skills and abilities necessary for professionals and engineers in the fields of Computer Science, Robotics, Artificial Intelligence (Miller and McBurney, 2008; Jacovi et al., 2021). Robotics as a learning tool focuses on the use of robotic platforms in educational settings (Li and Li, 2009; Bertacchini et al., 2010; Bertacchini et al., 2012; Gabriele et al., 2012; Gabriele et al., 2017; de Jong et al., 2018), while social robots have been used for subjects with particular learning disabilities (Simut et al., 2016; Scassellati et al., 2018). Based on constructivism theory (Piaget, 1970; Bruner, 1997; Rannikmäe et al., 2020), later developed by Papert (1993) and Resnick (Rusk et al., 2008) that envision the construction of knowledge as an adaptation to the real environment, a rich literature on educational robotics as a learning tool exists.

Leveraging students’ programming knowledge, using the Problem Based Learning or Project Based Learning (PBL) method and the NAO robotic platform, we built a class lab in which undergraduate students, from the Electrical Engineering degree, attending an Applied Mathematics course, solved real-world problems that may arise when the NAO is used in applied contexts (such as in Bertacchini et al., 2017). Working on authentic problems allowed the generation of solutions that amplified the potential of the NAO, making it suitable as a social robot, and thus transforming the system from unsuitable for real world application to capable of dealing with complex problems. In particular, students solved NAO’s locomotion problems, allowing the system to walk with an autonomous method, even if pre-programmed, problems of vocal and gestural interaction in order to interact with people socially, problems related to the recognition of people, which is done in this platform only through a complex architecture to be programmed in Choreographe. In fact, students solved such problems by interfacing the Choreographe platform with the Mathematica software and the Wolfram Language (WL). This approach allowed students to move from a linear programming model, to a multidimensional model of programming, while also connecting the system to a number of resources that Mathematica contains. The structure of the work is as follows. After this introduction, Section 2 gives a brief review of the functional programming realized by using the Wolfram Language. Section 3 reports on the subjects that attended the experimentation, the NAO platform and the Project Based Learning methods. Section 4 describes the students’ projects while Section 5 reports on the PBL assessment and the students’ qualitative feedback. A final discussion closes the work.

## Beyond Educational Robotics: Functional and Knowledge--Based Programming

The experimentation carried out in the educational robotics research lab concerns the use of some conceptual tools and the adoption of the Project Based Learning or PBL approach. The idea is to extend the potentials of the robot, which uses Choreographe software as a programming tool, with the powerful Wolfram language (WL) (Wolfram, 2018). WL is a symbolic programming language that uses symbols for computation, where symbols can be any kind of digital data. If appropriately encoded, these data are interpreted by the system as elements on which computational processes can be activated. WL is a knowledge-based environment, as it connects to the main scientific databases belonging to almost all scientific domains. It also contains a number of services that provide access to social media data. By embedding the documents, texts or other multimedia data contained therein into the notebook (the Mathematica working environment), these objects can be manipulated in various ways and calculations can be done on them. WL is a language of computation as it contains computable functions, and thousands of simulations of mathematical, physical, and chemical and/or biological processes. On the Wolfram Cloud (https://www.wolframcloud.com/) thousands of notebooks are also stored, which can be useful models that intercept the functions to be designed by students in this experimentation (Wolfram Cloud, 2021). There is also a “Wolfram Language Code Gallery” (https://www.wolfram.com/language/gallery/), a repository of reusable codes, through which, starting from the simplest codes, you can learn to program and design your own applications. In addition to the above, the WL incorporates a series of Artificial Intelligence tools such as Machine Learning and super functions, which allow realizing advanced functions such as computer vision, Big Data analysis, spoken language processing, face and gesture recognition, etc.

All of the students already had basic knowledge of C and C++, as typical programming languages for electronic engineers. In this research, the emphasis is not on procedural coding as such but on designing the social functions of the robot, using functional and rule--based programming examples (borrowed from the Wolfram Language), to be adapted to the design of a social robot with true real-life support and interaction tasks. The NAO robot has to move autonomously in the environment, to entertain with different types of users, to recognize and have a conversation with them (on specific topics), trying to identify hand gestures and the users’ emotions. The constructivist approach in Mathematica is Lego-like. However, instead of using bricks in the actual physical reality, as in the case of many educational robotics platforms, by using Mathematica students have to assemble conceptual bricks, taken from the Wolfram Language. This completely changes the approach of programming. Instead of thinking of the program as a sequence of actions to be embedded within the robot, the program will emerge from students’ mental representations of the functions the robot must perform in real life. The ability of programmers to develop accurate mental representations supports critical programming tasks (Petre and Blackwell, 1999), helping in formulating problems. Some of these aspects were investigated to get student feedback on the cognitive processes employed in WL, compared to traditional programming languages.

## Subjects, the NAO Robot and the Project Based Learning

### Subjects

18 students, 17 males and 1 female (average age 22 years), of the Electronic Engineering degree, attended the experimentation, in the course of Applied Mathematics, as a theme to choose from the various options the teacher had given. They worked from November to December intensively, supported by a researcher, who followed all the development of the design of the social robot, using the NAO Robot at the laboratory of Cognitive Science and Modelling, University of Calabria.

### The NAO Robot

**FIGURE 1:**
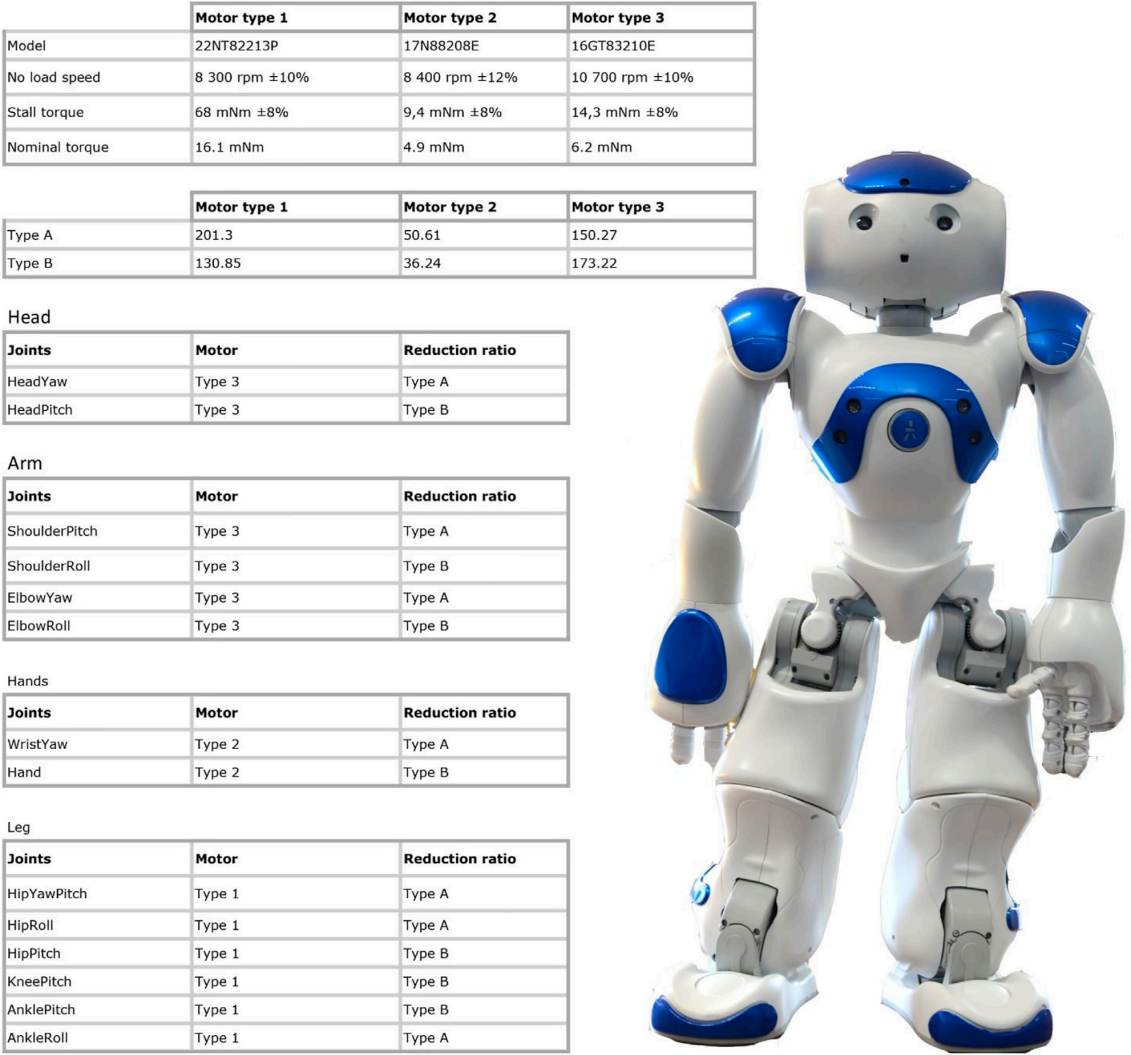
Technical specification of the NAO Robot. It is one of the most advanced humanoid robot on the market. It is a programmable, open source, 58 cm tall robot.

The version we used is from 2016, NAO Next Gen, with an onboard computer based on ATOM processor, HD cameras and interactive capabilities: voice and gesture recognition. It has 21 degrees of freedom, 5 inertial central axes, ultrasonic proximity and underfoot pressure sensors. It is equipped with a multimedia system consisting of 4 microphones, 2 loudspeakers and 2 CMOS cameras. For further details on motors and speed, see Figure 1 in the box.

### A Short Background About the Project Based Learning Approach

Developed about 30 years ago in the medical sciences (Walker, et al., 2015), the Project Based Learning (PBL) approach is a method that allows students to work as if they were researchers, posing problems and trying to solve them (Savery, 2015). Unlike traditional classes, in the PBL approach, students are first made aware of real-world problems. Therefore, they must learn specific topics and acquire specific skills that are then used to solve those problems or accomplish the required projects. Therefore, the problems and the context in which those problems occur become the specific scope for teaching and learning. Attributes of the PBL approach were considered in Boud and Feletti (1997). Duch, Groh and Allen (2001) described the methods used in PBL, along with the skills that students gain, including the ability to think critically and solve complex problems, to find and evaluate information sources themselves, and to use their cognitive resources independently. In addition, through PBL students learn to work cooperatively, demonstrating communication skills, using content knowledge and intellectual skills to become recurrent learners. Torp and Sage (2002) instead describe PBL as learning organized to investigate and solve ill-structured real-world problems. According to these authors, students are problem solvers trying to get to the root of the problems and the conditions necessary for problem solving. Other authors assume that the problems to be provided to students should not have a single answer, so that students develop a range of possible alternatives (Hmelo-Silver, 2004). An interesting summary of the key features of this method is summarized as follows (Savery and Duffy, 1995):

1. Students must take responsibility for their own learning. It has been seen that motivation to learn accelerates when responsibility for problem solving increases.
2. Data presented must be poorly structured in such a way that this frees up critical thinking skills.
3. Learning must be multidisciplinary.
4. Collaboration and work in team is essential.

These features have been implemented in the PBL educational activities we are reporting on.

### The Project Based Learning Educational Activities

Task assigned to students concerns the development of a social robot using the NAO Robot (Figure 1). The system has to move in the environment in an autonomous way, avoiding obstacles, to interact with people by activating voice communication and making short conversations with different users, to recognize the faces and spoken language of the users, and a specific set of hand gestures.

**FIGURE 2:**
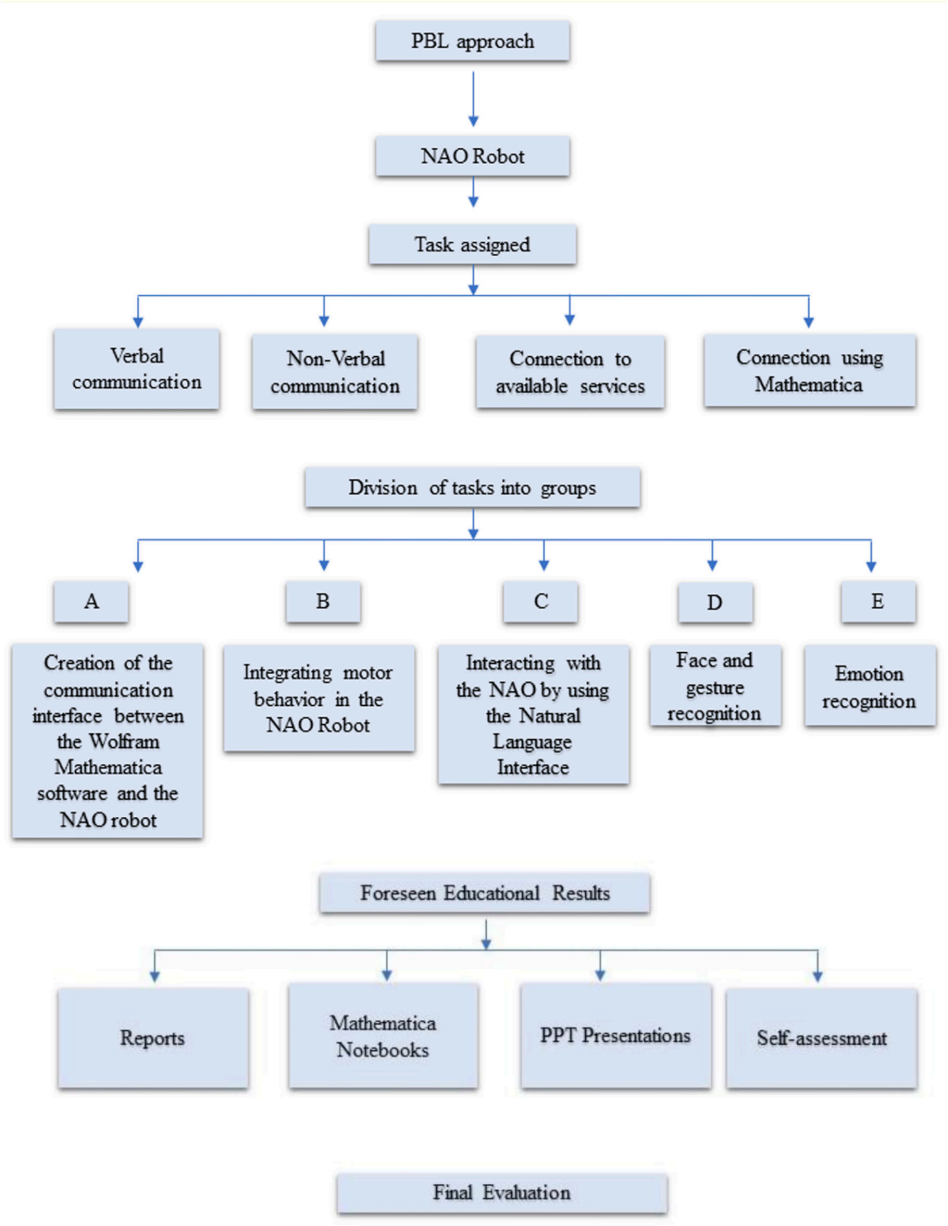
Step by step description of the PBL method.

The approach we used to implement this educational robotics activity is a PBL approach. We envisioned providing students with the typical researcher environment, allowing them to use the lab as a fish-tank of ideas to be developed within the WL. An outline of the PBL method used in this experimentation is reported in Figure 2.

Therefore, the basic architecture of the social robot to be developed concerns the behavioral modules focused on:

a. Verbal communication (recognition and production of spoken language)
b. Non-verbal communication of users (face recognition, recognition of hand gestures)
c. Connection to different kind of available services, including Social Media
d. Connection to the real world, by using the Mathematica Environment and the Wolfram Language principles

The idea is to connect the NAO programming environment with Mathematica and from this to a cloud, in order to speed up the processing of the information and use the knowledge-bases present within this system in order to create a real connection of system with the world. In particular, the tasks for each group are reported in Table 1.

**TABLE 1:** List of the projects assigned to the students.

Projects	Sub-project
(Group A) Project 1. Interfacing the NAO robot with the Mathematica programming environment, to perform user and environment interaction tasks	Develop the interface between Mathematica and Choregraphe
	Develop the cloud for allowing the storage and processing of information
	Develop the interchange format to be used for all groups
(Group B) Project 2. Developing the robot motor-behaviour, providing it with trajectory commands or leg and arm movements generated by Mathematica programming, by stowing behavioural routines in a database, and recalling them in situations of interaction with the environment and users	Develop the NAO’s motor behaviour
	Develop the obstacles avoidance system
	Develop the processing of trajectories for allowing the system to move independently in the environment
	Develop a set of trajectories already used
(Group C) Project 3. Activating the NAO as a conversational interface to allow interaction with the users	Develop the NAO’s verbal communication (recognition and production of spoken language)
	Develop the storage of possible sentences that is possible to reply with to a conversation input, coming from humans
(Group D) Project 4. Programming the facial and gesture recognition systems	Develop the face recognition system
	Develop the gestures recognition system
(Group E) Project 5. Programming the emotions recognition system	Develop the emotions recognition system
Programming the display of the NAO postural emotions	Develop postural emotions
Project 6. - All groups - Reviewing the work of other research centres that are using the NAO platform for similar educational activities	Find relevant scientific literature on the NAO robot
	Find repository of already developed projects with the NAO robot that can be useful for all groups

**FIGURE 3:**
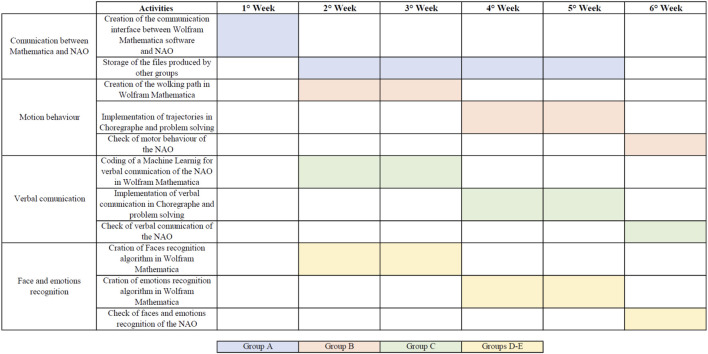
Work time and organization of PBL phases. Groups worked for a total of 6 weeks, from the first of November to mid-December.

The working time of the groups is exemplified in the diagram that is shown in Figure 3.

Regarding the role of the teacher, in the realized PBL experimentation, instead of the usual transmission-oriented approach, an interaction-oriented perspective was preferred to improve students’ computational skills The goals of PBL are to change the role of the teacher from the traditional transmission-oriented perspective to an interaction-oriented tutor role. Therefore, in the Educational Robotics setting implemented, the teacher was given a guidance role, within constructivist environments (Chevalier et al., 2022). In fact, a researcher worked with all the groups in order to facilitate communication and the sharing of coding implementations as the educational activity was running on. She used journals for each meeting to record the groups’ various experiences during the educational activities. The analysis of the students’ programs showed that the planned functionalities for the NAO robot were perfectly implemented and embodied in the system. All the students used the WL main features, adapting their programming styles from sequential programming to a knowledge-based approach. The laboratory environment and the researcher that worked together for the NAO behavior implementation improved the learning of the Wolfram Language potentials and the communication among groups.

To understand the cognitive shift from traditional programming language to the WL, the following questions were asked to the students during the lab activities:

a. What is your opinion about the swift from the sequential programming to WL?
b. What kind of cognitive processes are deployed in WL programming?
c. According to your opinion, what is the role of mental representation in WL?
Feedback on these questions will be given in Section 5.

## Description of the Students Projects

In what follows, we report about the details of what the students did for solving the problems of the NAO as a social robot.

### Group A. Creation of the Communication Interface Between the Wolfram Mathematica Software and the NAO Robot

**FIGURE 4:**
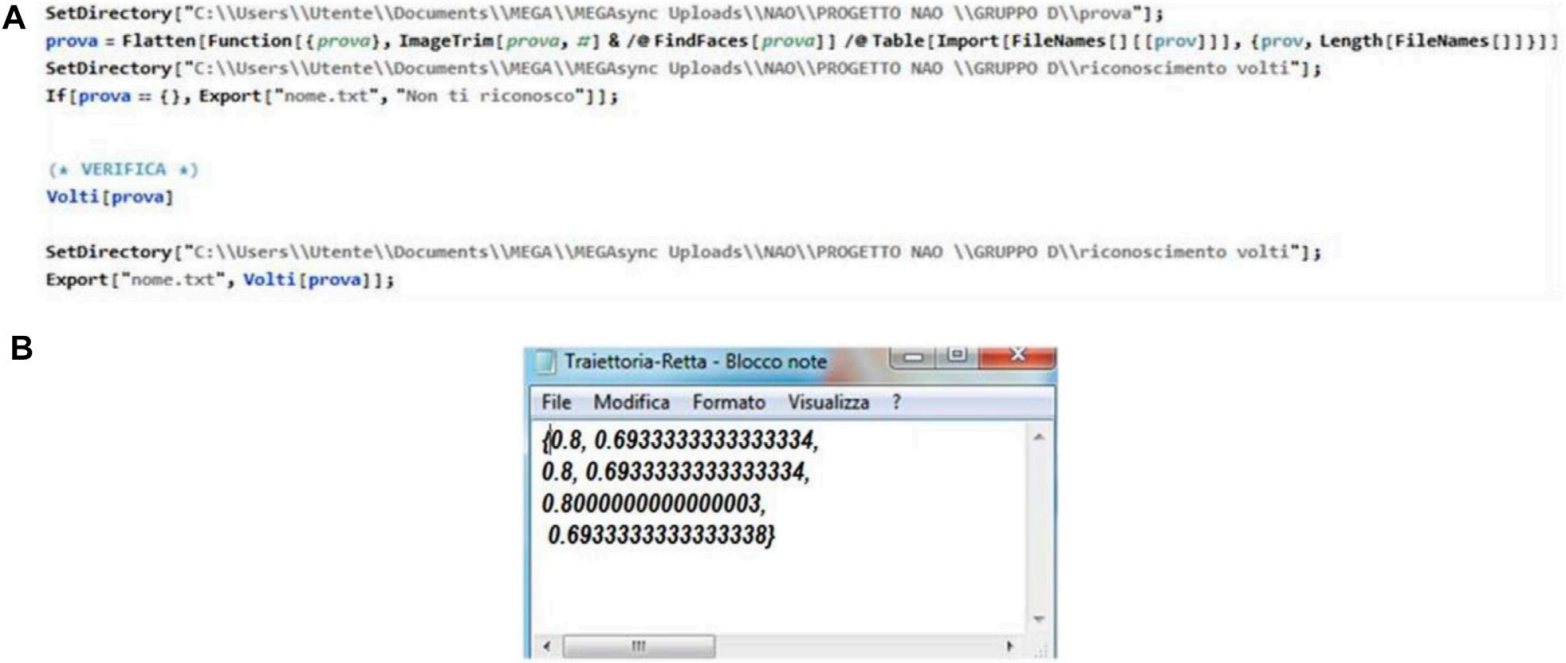
**(A)** Extracts of the text files that are used for the functions of text to speech, face recognition and the **(B)** motor trajectory the robot must perform.

The development of this function was initially particularly complicated, since there is no software capable of interfacing the two programs dynamically. To implement this function, students used a cloud on which to place the data produced, making it available for further processing. In order to realize this function, the students used three main software. All calculations and elaborations were performed within the Wolfram Mathematica software, while for the final interface, they used the Choregraphe software, being a programming environment already set up for the communication with the NAO robot. Finally, Filezilla software was used for synchronizing the server and the cloud. In order to allow the communication between the NAO and the Mathematica software, three different learning machines were built in Mathematica for three different operations: 1. recognizing the people the NAO meets when interacting with the group of students, 2. answering questions posed by the students to allow an initial social dialogue, and 3. recognizing the main non-verbal gestures each student makes. Furthermore, the import and export functions were also included in order to send the contents used to communicate with the robot to the Choreographe environment, usually realized by a. txt files that the NAO is capable to read for enabling the text to speech function, but also to communicate the points of a motor trajectory the robot has to follow, to recognize the specific person the robot is interacting with, by looking into its own camera, or the gesture he/she is actually doing (Figure 4).

**FIGURE 5:**
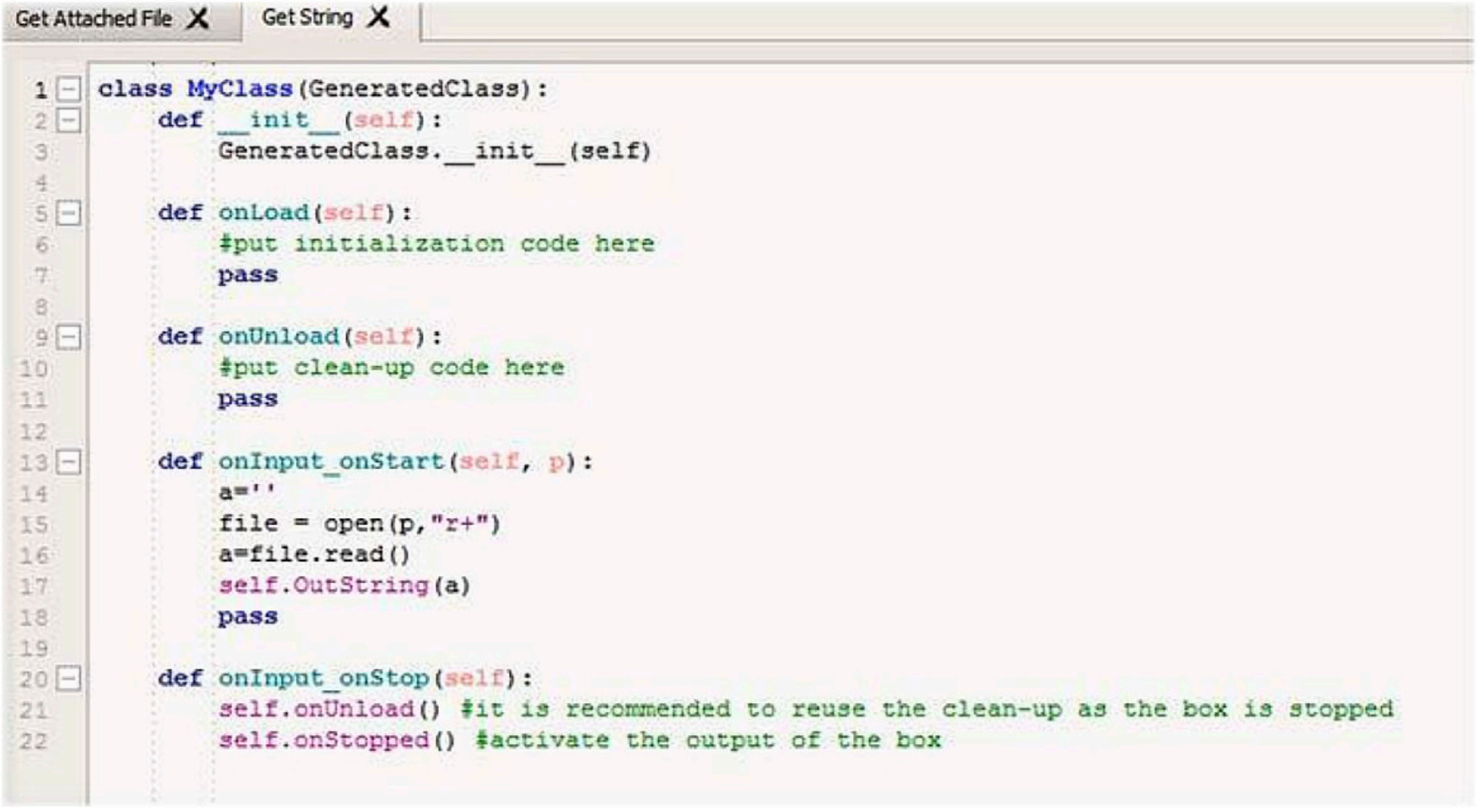
In this figure, the code of the script, modified with Python, used to read a. txt file is represented. The robot receives it as input, uses the function “open” to open the file and then the function “read ()” to read it and pronounce vocally the string containing the text of the file.

To allow the Mathematica and Choreographe dialog, it was necessary to program and modify some blocks of Choregraphe with the Python programming language. This operation has been necessary so that the blocks are able to receive these files (.txt), interpret them and provide adequate instructions to the NAO. These blocks were connected in a proper way to the blocks already present in Choregraphe so that the robot could execute the “face detection” function, take a picture through its camera, locate the sound and record what it hears in order to save data permanently and, finally, to read a string in the text file and reproduce the text vocally. The created blocks are then placed in the MEGASync folder, specific to each function, so that they are automatically activated at the proper time (Figure 5).

**FIGURE 6:**
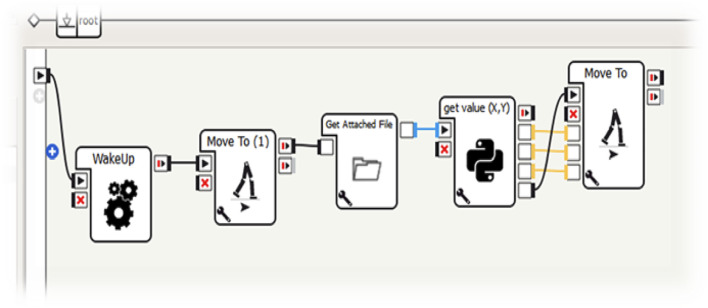
Sequence of commands used to activate specific trajectories of the NAO robot.

The script code used for the movement reads from the text file a list of data points in an ordered way (i.e. x , y and θ ). Then three lists are formed with three distinct cycles, scrolling the list with different steps so as to take all x , all y and all *θ*.The index of the formed lists is the point number, i.e. (first point, second point, etc.). These coordinates are passed neatly into the point-by-point output with a parameter-adjusted delay and are sent to the appropriately modified “Move To” block to receive the externally supplied point coordinates rather than from parameters (Figure 6).

However, it remained to solve the problem of synchronization between when the NAO acquired the information directly (photos and audio recording) and the output necessary to perform the actions synchronously. This function seems a fundamental feature for a social robot. For this reason, the MEGASync cloud was used in order to have the transmission of files from one software to another in a synchronized way. On the cloud, the students created a folder with all the files used in the execution of the project and this in turn is synchronized with the remote FTP server of the NAO, in order to capture the required files and save them in a local folder. Each group of students had its own folder, connected with all other folders, in order to save all the progress of the project, to make all the functions accessible to the system.

**FIGURE 7:**
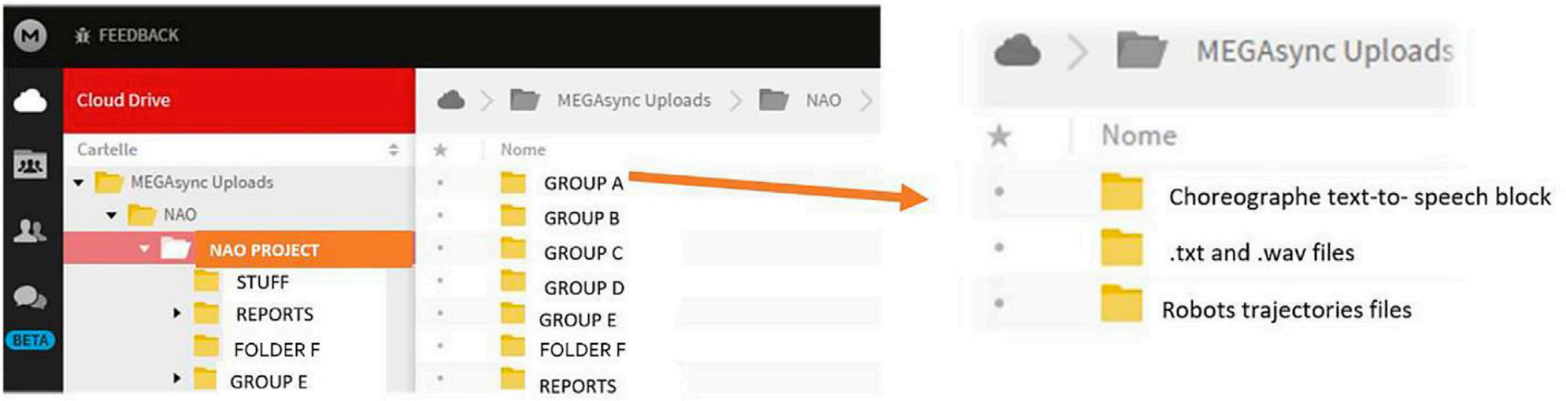
Integration of the communication modules realized by the students of group A. As it is possible to see, the system allows the NAO Robot to interface with the Choreographe module, to record texts and sounds, to perform motor trajectories.

To synchronize the remote folder to the local MEGASyn one, students searched for a software able to automatically perform the synchronization between the two folders. Figure 7 represents the communication diagram developed by the students belonging to Group A. Inside the MEGASync system, the interfacing system allows the information to arrive dynamically to the NAO and to be able to manage them as if they were in real time. This approach has allowed a very interesting behavior of the NAO, correlated to the timing of the interaction that we usually have between human subjects.

**FIGURE 8:**
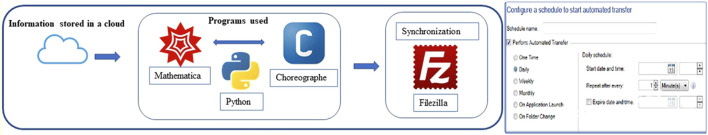
Filezilla module to configure automated transfer between the different files in order to allow synchronization in the robot actions.

However, with the more appropriate application, it took more than a minute between actions. Therefore, students chose to do the transfer manually, by using the Filezilla program, by double-clicking on the file in the remote site that it was necessary to transfer. Figure 8 contains the configuration module that it was possible to activate with different kind of automated transfer set-ups.

The results in terms of computational skills of the students in this group are related to the dynamic interfacing of the two programs (Mathematica and Choreographe) using the NAO robot as a communication interface.

The students of this group built the following functions:

1. Develop the interface between Mathematica and Choregraphe;
2. Develop the cloud for allowing the storage and processing of information;
3. Develop the interchange format to be used for all groups.

### Group B. Integrating Motor Behaviour in the NAO Robot for Allowing an Autonomous Interaction With the Environment

The work of Group B aims to implement the robot motor behaviours based on mathematical knowledge. The implementation of the code and the related computations have been managed externally with the Mathematica software. The results of these computations have been shared on the server cloud, to which the robot interfaces, thanks to the application developed by Group A that connects Choreographe to Mathematica. Specifically, Group B dealt with the movement in space and the interaction of the robot with the environment. The movement is realized using a discrete set of points extrapolated from mathematical functions. By interaction with the environment, instead, it is meant the ability acquired by the NAO (through appropriate commands) to prevent collisions with any external objects that interfere with its movement.

Choregraphe, the default software for working with the NAO robot, does not offer the ability to choose trajectories resulting from mathematical functions. Instead, programming the NAO with Mathematica software allows the robot’s ability to move through its environment to be enhanced, providing it with the dynamism and fluidity of movement that humanoid robots usually lack. Choregraphe uses a block programming approach, editable with Python; then, by interconnecting the various blocks, it is possible to give the NAO a command to perform a specific action and a series of actions to realize movements that are more complex.

To provide the robot with a mathematical function to serve as a motor trajectory, it is necessary to treat the trajectories with Mathematica, discretizing the known functions to be represented and saving the points obtained in lists that will then be provided in the form of text files to the robot. If we want Choregraphe to interpret the lists of points, the robot must receive commands to reproduce the desired trajectories.

**FIGURE 9:**
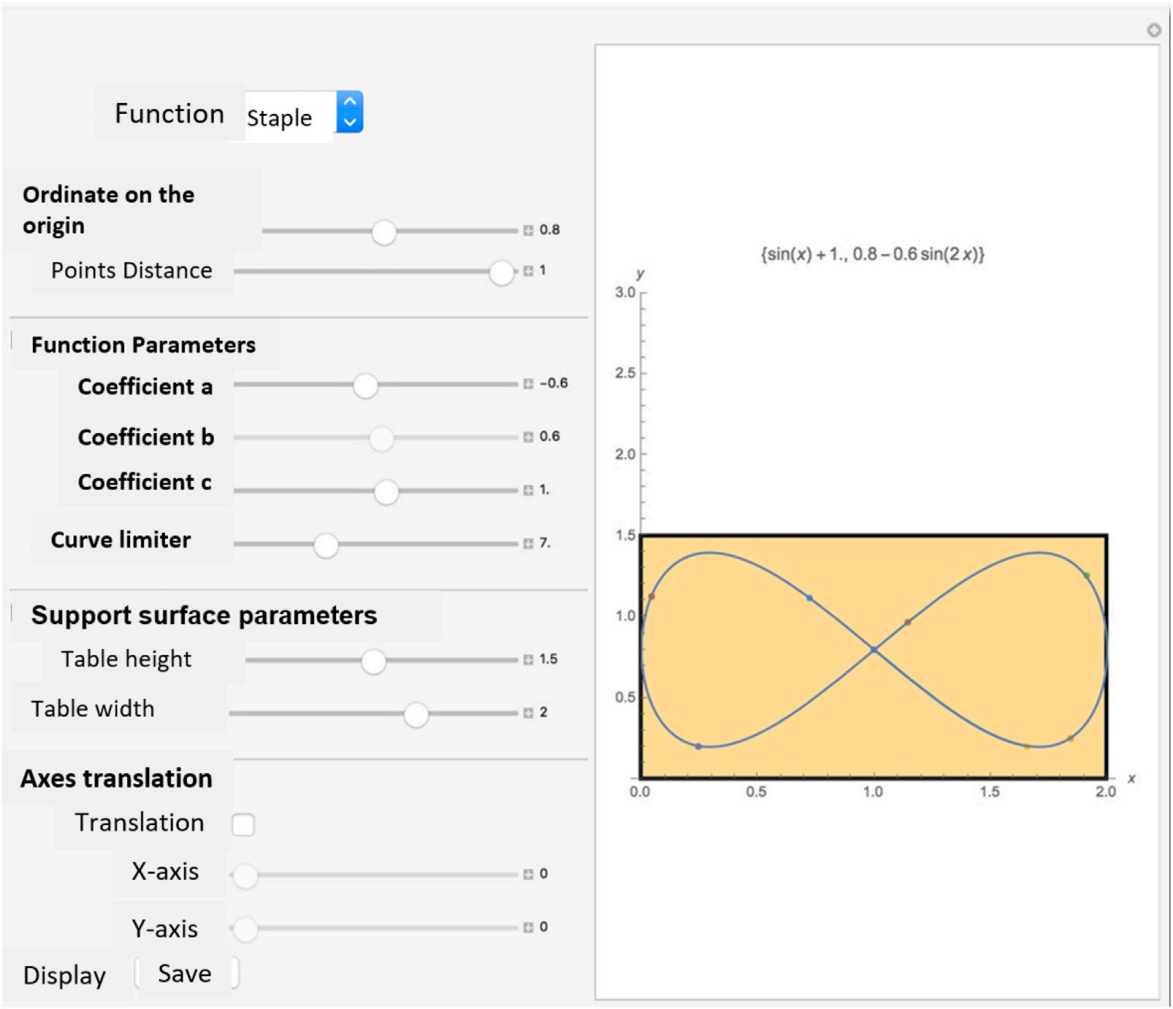
The Mathematica interface to plan the motor behaviour of the robot.

Specifically, the implemented process consists of the following steps:

1. Choose a specific function and represent it in Mathematica using the Plot command;

2. From the trajectory, using commands such as ListPlot and Table, points are extracted in order to qualitatively approximate the behaviour of the function;

3. The considered points are exported and saved in a text file, following a precise formatting (standard defined together with the interface Group A).

4. The text file will then be provided to Choregraphe, in a block previously programmed that accepts and interprets the given file type with the proper internal formatting. At this point, the NAO is able to reproduce the planned trajectory. In Figure 9, the Mathematica interface to plan the motor behaviour of the robot. The program has a clear and simple interface, making it easy to use even for less experienced users. Below is an explanation of the various graphic elements and parameter controls.

The yellow box represents the working plane, which corresponds to a reproduction of the real and unobstructed space within which the NAO is free to move. The dimensions of this “plane” can be modified and better adapted to the real conditions of the physical environment in which the NAO is located using the sliders indicated as “Support surface parameters”.

The PopUpMenu offers the possibility to choose the function to represent. In the specific case, the choice is between straight line, sine, and staple. The presence of some parameters makes it possible to control the geometry of the function. In particular, it is possible to modify:

1) the ordinate at the origin, which allows choosing the ordinate from which to “start” the function; 2) the distance between points, which allows to correct the distance between the various points to be exported in order to discretely recreate the function; 3) the coefficients a, b, c, which allow the slope, phase, amplitude or “geometric” characteristics to be modified in relation to the selected curve; 4) the curve limiter slider, that allows to control the maximum value that x can take in the graphical representation. In practical terms, this means that it is possible to “shorten the curve” in order to avoid, that it protrudes from the working plane.

At the interface level, the presence of two buttons that return a control on the coordinates of the curves treated allow for the following functions:

Visualize, that prints in a pop-up window two matrices containing respectively the x−y coordinates of the points and the x−y coordinates translated with respect to the origin with the corresponding angle of inclination of the vector;

Save, that saves the list of points assuming that the point preceding every other point is, each time, the origin of the axes (each point is represented as the distance along the x−axis and y−axis with respect to the previous point.

**FIGURE 10:**
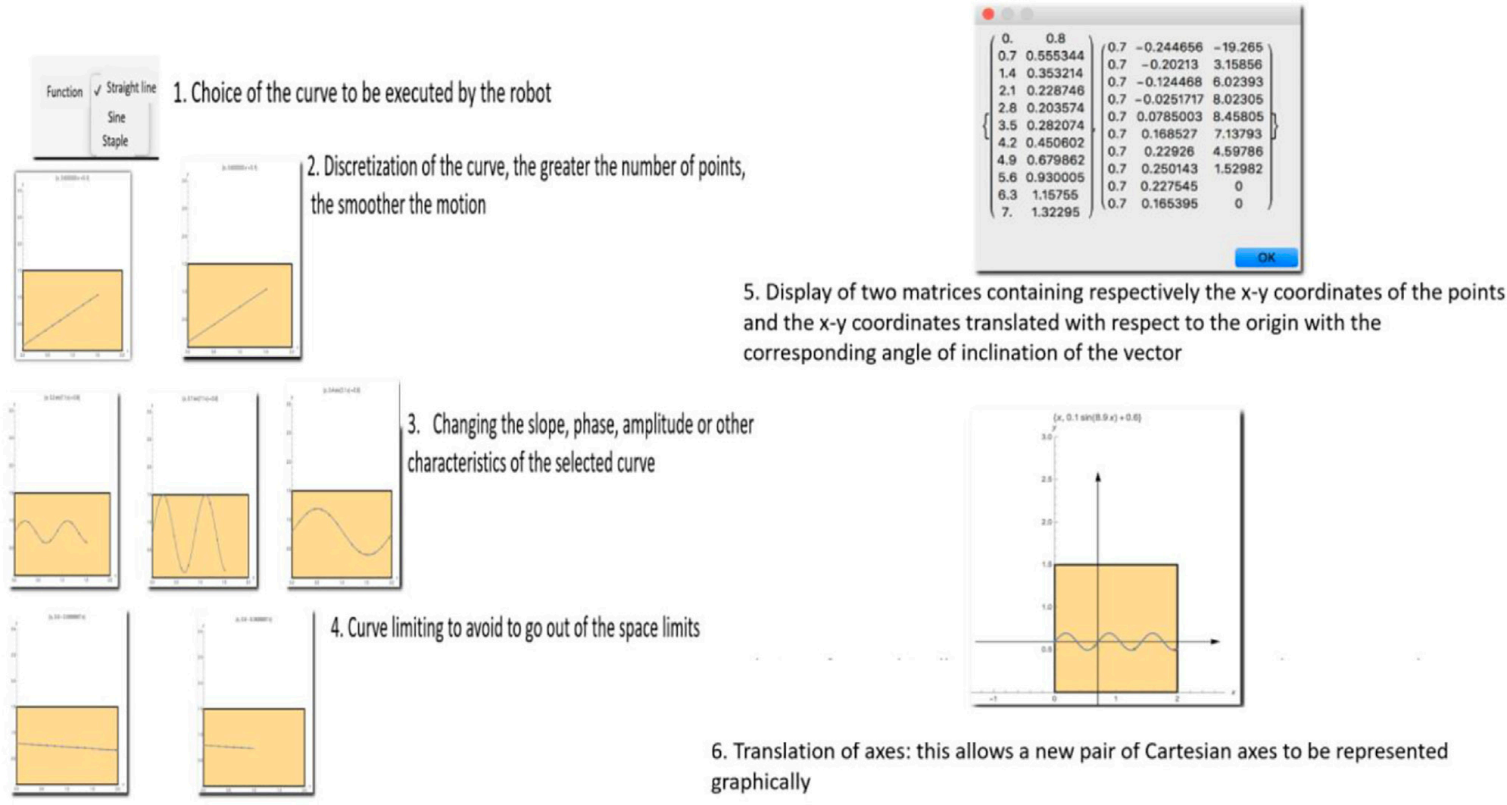
The main function of the interface to create mathematical trajectories for the NAO robot.

Translation of axes, function that allows a new pair of Cartesian axes to be represented graphically, useful for visualizing at a glance the position of each point with respect to the new reference (origin of the translated axes). Furthermore, by using the sliders, it is possible to control the disposition of the axes on the plane (Figure 10).

The fundamental problem of Group B concerns how the NAO moves from one point to another. After saving the points of the function, the robot must interpret the points and their location in space.

As the problem has been defined, the NAO cannot interpret a mathematical function and move along that trajectory automatically. In fact, it is necessary to provide the robot with a list of points extrapolated from the trajectory: only by indicating the points, the robot will know how to move. By following an iterative process, structured in finite actions, the NAO receives a point, performs the move, and finishes its command action. For the next point, the robot will restart the motion considering only the new point. This means that, having no “memory” of the past trajectory, since each movement is independent and does not depend on the previous ones, at each step the robot will move towards the indicated direction, considering the origin as its starting position. However, this does not correspond to the function of an intelligent robot. If the NAO must move from point 4 to point 5, for example, it will move not by 1 but by 5 (since point 5 will be understood as the vector from 0 (current position) to 5.

To solve the problem it was necessary to translate each point so that if, for example, the NAO is at point A and has to go to point B, the coordinates of point B exported are not referred to the origin but are equal to the distance between A and B. At the implementation level, a FOR CYCLE with appropriate indices was used, to subtract the previous point from each one. An analogous discourse is valid for the calculation of the angle curvature that the robot must assume while travelling along the segment AB.

Choregraphe is used for interfacing the NAO with Mathematica and, in this specific case, to interpret the list of points created in Mathematica.

**FIGURE 11:**
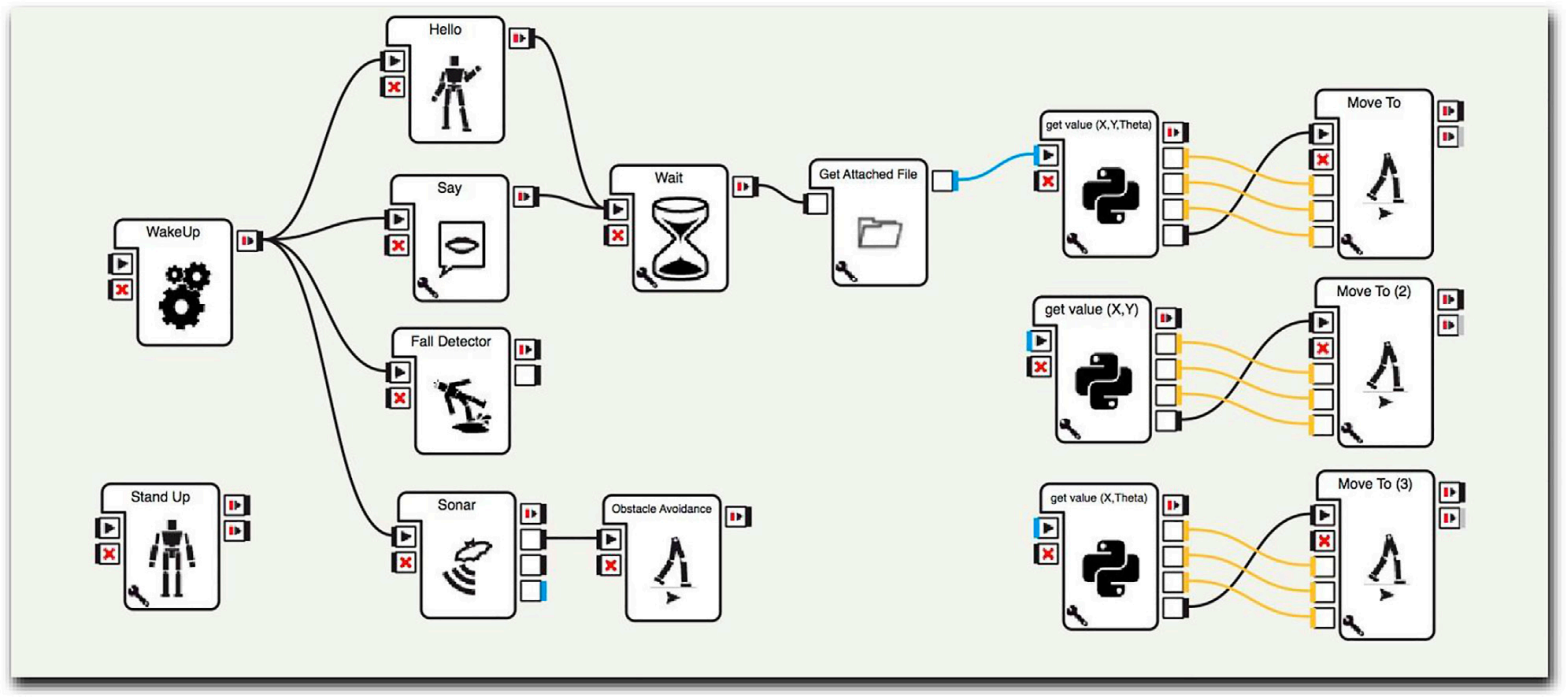
NAO activation procedure through Choreographe.

To allow the NAO to perform the chosen trajectories, it is necessary to alert the NAO with the appropriate “WakeUP” block. The program built on Choregraphe foresees that, at this point, the robot greets with the right hand and explains what it is about to do: “Hello I am the NAO Robot, now I will execute a trajectory imported from Mathematica”. This first set of commands was not necessary, but it was included to make the presentation more pleasant and appealing (Figure 11).

Subsequently, the NAO retrieves the file Trajectory. txt (in which the list of points exported from Mathematica is saved) from the program folder by using the command block GetValue (x,y,θ) (block wisely created by the members of group A). ON receiving this command, interpreted with the right formatting, NAO will start to move thanks to the “MoveTo” block.

**FIGURE 12:**
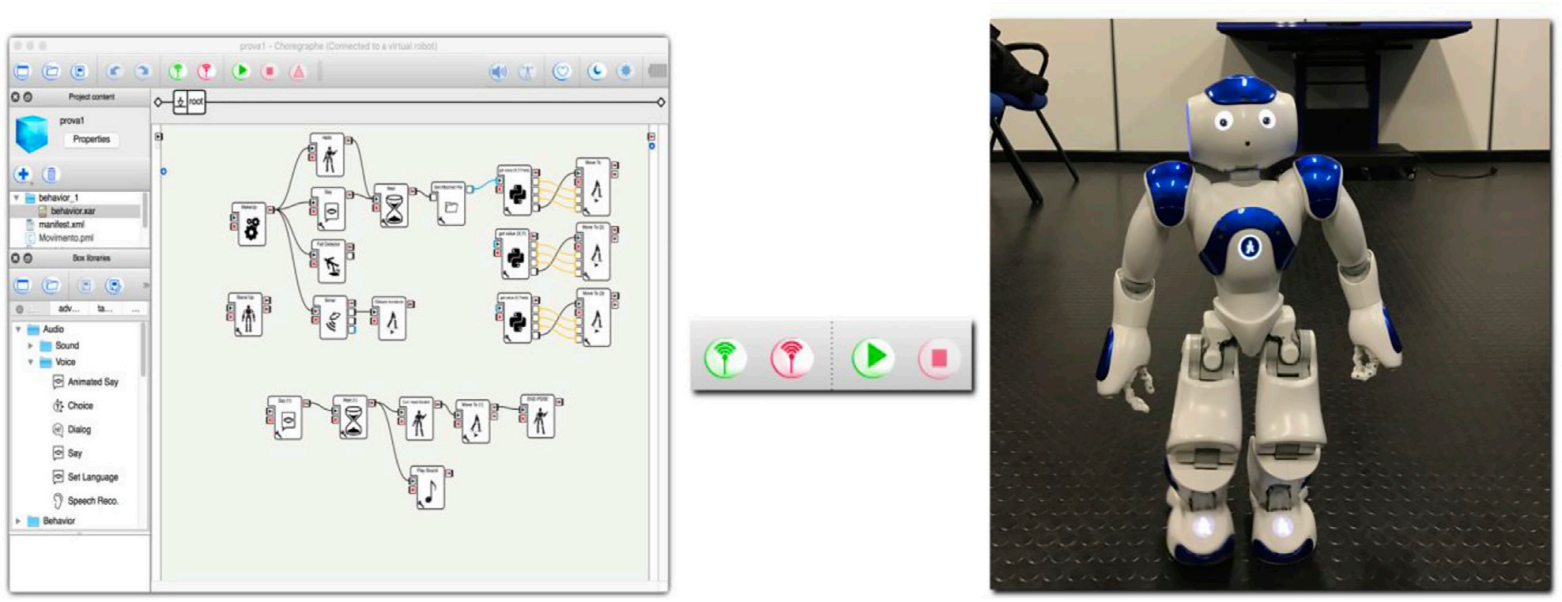
After having created the trajectory file in Mathematica, and having closed the program, the interface with Choreographe creates a movement. pml file. This file, if opened, allows the NAO to create the specific trajectory by activating the green play button.

It is important to underline the importance of the process of the Choregraphe block that makes possible the translation of a simple list of numbers into a “sensible” succession of points, characterized by x-coordinate, y-coordinate and angle. In parallel to these operations, the “Fall Detector” and “Sonar” blocks have been introduced. These commands, exploiting the complex sensoristics of the NAO robot, protect the integrity of the robot preventing it from falling or bumping into an obstacle. It is easy to prove the usefulness of these commands. When the robot finds an obstacle along its trajectory, it will stop, avoid the obstacle and, eventually, continue its trajectory (Figure 12).

The students of this group performed some simulations with the NAO. We report a short synthesis.

Many problems encountered in the development of mathematically based trajectories are related to how the Mathematica and Choreographe software interfaces with NAO and how NAO interprets and processes the data provided. Examples of first approaches and related problems encountered will be given on what follows.

In the first test, having chosen the equation y = 0 (horizontal line), limiting the graph to x = 2 in order to have a displacement of 2 m (without this measure, the displacement would have been infinite since the line is infinite), four points of x = 0.5, 1, 1.5, 2 have been extrapolated from this line. The NAO instead of traveling 2 m has travelled 0.5 + 1 + 1.5 + 2 = 5 .

In the second test, the equation y=x with x limited to 1 has been chosen. As a result, the NAO has moved diagonally as expected. However, the robot has again travelled more meters than it should have.

The problem that has been detected is that the Robot, once the current displacement process is finished, is not aware of the previous displacements. It treats each displacement separately, interpreting, at the beginning of each movement, its position as if it corresponds to the origin. Consequently, by giving as first point A(0,1) and second point B(0,2), NAO will perform two movements whose result will be a displacement of 3 m along the x−axis .

The students found a solution. Instead of transmitting to the NAO the coordinates of the points as printed on the Cartesian plane, it has been necessary to transmit the dx and dy variation between one point and the next.

In the third test: after having made the correction (i.e. the points are all shifted with respect to the previous point intended as origin), the trajectory of an inclined line of a certain length is re-executed.

As a result, the NAO perfectly executes both for the distance covered and for the inclination of the line itself.

In the fourth test, the students changed the trajectory. The NAO has to perform a sine trajectory.

As a result, the robot moves laterally, not quite correctly. In this case, the NAO interprets correctly the list of points (there is no mathematical-geometric problem), but the movement performed does not correspond to expectations (the problem lies in the interpretation of the data provided by the robot).

The solution is to use the block “MoveTo” of Choregraphe that requires, in addition to the parameters x and y , the addition of the parameter θ , which provides the angle of curvature to the robot trajectory.

In the fifth test, the students tried the updated version of the program in which the angle is included among the parameters required for the execution of a straight line. As a result, the robot at each point also updates the angle; consequently, it runs a circle instead of a straight line. For example, if the straight line had been inclined by 30°, and, therefore, the coordinates were of the type ( x,y,θ ), NAO, at each update of the parameters, would have inclined 30° more than the previous section.

By including the addition of the angle, the sixth test provides a sine trajectory. In this test, the robot moves in a similar way to the previous case, with an incorrect trajectory.

The problem detected is related to the behaviour of the coordinates, also the angles “add up” at each step. A possible solution foresees to proceed by “subtracting” the previous angles from the following ones, likewise to the solution of the first problem.

In the seventh test, the students tried the revised code. In this case, the robot performs the movements correctly and moves naturally along the trajectories. However, the NAO did not understand properly angles. Therefore, the exact behaviour is reduced to only a portion of the trajectory. After making a movement, the robot forgets its previous position. Therefore, to the problem of choice at each point of the reference system, is added the problem of interpretation of a reference point, rotated with respect to the origin. In other words, if at the first step the NAO moves along a straight line inclined by 45°, it will correctly execute the trajectory. On step two though, it will re-execute a new rotation of 45°, nullifying the correct trajectory. A possible solution is to apply an algorithm that allows the export of the points in Mathematica in order to prevent the error and its propagation during the robot behaviour.

The results in terms of computational skills of the students in this group are related to the use of the experimental method to test the motor performance of the NAO Robot. In addition, the work of building trajectories in Mathematica, allows for the creation of a series of paths, in known environments, making the NAO very skillful at motor management, by avoiding obstacles in them to best perform the designed tasks. This could allow the NAO to be perfectly adapted to living environments.

### Group C: Interacting With the NAO by Using the Natural Language Interface

The task assigned to team C is to manage the dialogue of the NAO, humanoid robot. All the processes of dialogue analysis, processing of responses according to the inputs and the output of verbal statements produced by the robotic system, are performed externally by integrating both Choreographe and Mathematica programs, shared with the robot through an ad hoc developed cloud.

Specifically, the task of this group allows the recognition of some statements during the interaction with a human subject who asks something to the robot, with its response appropriate to the context and the request made. A series of methods in Mathematica made it possible to capture voice data, analyse it and allow the robot to hold a simple dialogue with its human interlocutor.

**FIGURE 13:**
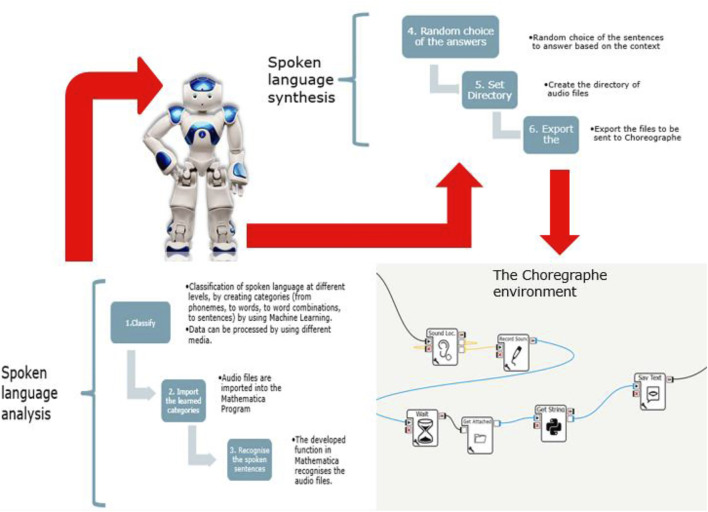
The process implemented by the students to allow a direct interaction with the users.

The role played by Mathematica is very important as it enables the automation of a basic process of dialogue, namely the understanding of what the human subjects say to the Nao. To do this, a Learnig Machine is created to recognise sounds (Figure 13).

In Figure 13, the steps of the process of recognition of the sentences, pronounced by a human subject and the answers provided by the NAO are represented. 1. The main command used is “Classify”, through which learning classes can be created, and thus the dialog addressed to the Nao can be distinguished. 2. Next, it is necessary to choose a folder from which Mathematica will load the audio file recorded by the Nao. To do this, the commands “SetDirectory” and “Import” are used. 3. When the program is started, Mathematica recognises the sentences by means of the previously created function “Text1”. The result is assigned to the variable out1. 4. Using the commands “Switch” and “RandomChoice”, NAO Robot is able to randomly choose one of the answers pre-set by the user corresponding to the variable assigned to out1. 5. Once the random answer has been chosen, using the “SetDirectory” it is possible to create the directory of audio files. 6. The, by using the command “Export”, Mathematica will save a. txt file, containing the answer, in a folder chosen by the user, that will be sent to the Choreographe application in order to be spoken by the NAO Robot.

The main command used is “Classify”, through which learning classes can be created, and thus the various questions addressed to the Nao can be distinguished. Next, it is necessary to choose a folder from which Mathematica will load the audio file recorded by the Nao. To do this, the commands “SetDirectory” and “Import” are used. When the program is started, Mathematica recognises this sentence/question by means of the previously created function “Text1”. The result is assigned to the variable out1. Using the commands “Switch” and “RandomChoice”, Nao is able to randomly choose one of the answers preset by the user corresponding to the variable assigned to out1. Once the random answer has been chosen, using the “SetDirectory” command and the “Export” command, Mathematica will save a. txt file, containing the answer, in a folder chosen by the user.

The Classify method can be used with almost any digitised data, including numeric, text, sound and image data, and even combinations of such data. The examples to be provided to the classifying machine may be either a single item of data that is to be classified, or a list of items, or an association of items, or the entire data set that may be provided. The functions that are usually used are expressed in the following graphic form: Classify [training, … ], training can be a Dataset object.

• Classify [training] returns a ClassifierFunction […] that can then be applied to specific data.
• Classify […, data], data can be a single item or a list of items.
• Classify […, data, prop], properties are as given in ClassifierFunction. This last function, generated by Classify, classifies data into classes. The main classification functions concern the following list (extracted from Mathematica, 2018; and shown in Table 2):

**TABLE 2:** List of functions generated by the Classify algorithm in Mathematica.

Decision	Best class according to probabilities and utility function
TopProbabilities	Probabilities for most likely classes
TopProbabilities	n probabilities for the n most likely classes
Probability class	Probability for a specific class
Probabilities	Association of probabilities for all possible classes
SHAPValues	Shapley additive feature explanations for each example
Properties	List of all properties available

The use of the “Classify” command is very intuitive and immediate. In order to have the sentences recognized, we have associated the sentences (indicated in the program with the generic term “Text”) to the Classify command. At the second level, we have associated the sentence components (nouns, verbs, other). At a more molecular level, the classifying machine was trained on the individual words, assigning to each word the series of alphabetical symbols that constitute it. After this classification of phrases, elements that constitute the phrases, elements that constitute the individual words, we import into Mathematica the various files that will compose the learning classes for the robot NAO. These files can be of any type, a letter, a number, an image or just an audio file. In order to allow Mathematica processes to interface with the NAO, it was also necessary to create the program in Choregraphe, which in turn must allow the recording of the desired question and the execution of the corresponding answer, pronounced by the NAO. Through the “Sound Location” and “Record Sound” blocks, the NAO records an audio track that will be saved in a folder of your choice using the “Get Attached File” command. After processing the data, Mathematica will return a. txt file containing the answer, which will be read through the “Get String” block and played by the NAO through “Say Text”.

Choregraphe uses a block-based programming, allowing for the connection of the various blocks among them in order to create the NAO behavior. Choregraphe program has a very important role in the spoken language synthesis. Through the commands “Sound Location” and “Record Sound” the NAO records an audio track that will be saved in a folder of your choice through the command “Get Attached File”. Choregraphe in this part of the project allows the recording of the desired question and the execution of the corresponding answer by the NAO. Through the “Sound Location” and “Record Sound” blocks, the NAO records an audio track that will be saved in a folder of your choice through the “Get Attached File” command.

After processing the data, Mathematica will return a. txt file, containing the response, which will be read through the “Get String” block and played by the NAO, through “Say Text”. Students encountered a number of problems inherent in this delicate phase of linking the NAO and Mathematica. In particular, it was impossible to insert all of the linguistic variables that the NAO must recognize in the same notebook, since the Mathematica software presented strange “crashes” and error messages, not syntax errors, but probably due to low computational capacity and therefore not due to incorrectly written code. In addition, the addition of more than 8 audio files for each variable can bring various slowdowns and “crashes” to your computer. Also, Nao does not record good quality audio tracks. All this could make difficult the recognition by the Machine Learning of the class of the audio file that the NAO must recognize and to which it must give a response, causing a bad understanding of the dialogue.

This group, in addition to providing remarkable results in NAO performance has developed a number of computational linguistics tools, which Mathematica software makes available. The Wolfram language not only has multilingual dictionaries, but also a dense network of structural semantic and word usage information that thereby amplifies the NAO robot’s comprehension capabilities. By manipulating the strings through the symbolic computation that the Wolfram language allows, along with the visualization systems that the system possesses, one can provide the NAO with data in an optimized way that improves performance in social interaction, providing a unique and powerful platform for natural language computation.

### Groups D. Face and Hand Gestures Recognition

The recognition of the face and especially the recognition of the emotions that the face expresses has long been a subject of study since Darwin. He began to pose the problem if precisely the expression of emotions is a universal model of behavior and, as such, expressed and understood in the same way in all human populations, regardless of where the different human populations have settled. After him, several authors have started to study the problem (Ekman and Friesen) and in very recent times it has become an important research topic in the fields of computer vision technologies. Robots encompass processes for recognizing both people’s faces and the emotions they express. In addition, these processes link to nonverbal communication in interaction with human subjects, including body postures and hand gestures.

In order to create a social robot, it is very important to have the ability to recognize humans living in the same environment of the robot. This is especially true in case of companion robots. They must be able to recognize the emotions that humans express in order to meet their needs, especially if such robots have the task of taking care of elderly people and children.

For allowing the face recognition, the students realized a machine learning able to recognize human faces, using three different software: Mathematica to program the machine learning, Choregraphe to command the robot and Mega cloud for synchronization and storage of files.

First, they had to acquire a photo archive by creating directories on the cloud; each folder was renamed with the last name of a member containing the respective “close-ups”. Each photo was cropped to exclude any errors that the software could have detected as a face and distorted the evidence.

Then the students started the programming on Mathematica, creating as many blocks as the number of students to be recognized, corresponding to the number of the directories on the cloud.

Using the “SetDirectory” command, they imported, via the file path, the photos of the members into their respective blocks and performed the face capture procedure with the “FindFaces” function; this command identifies and reframes the face of the person in the photo. The students used the member’s last name as the identifier of their block. There is also a block (null) containing random photos of people that NAO does not recognise so that if a stranger shows up, the robot does not associate her face to one of the existing blocks. The robot labels this outsider, as “I don’t know you”. Once the acquisition of the images was finished, the students created the learning set using the “Classify” command that associates to each block the name of each member of the group that must be recognised. To test the performance of the robot, students imported other photos, testing its correct operation. The first tests were inaccurate because the set had a reduced number of photos. Therefore, the students acquired new images from the same subjects to be recognised, increasing considerably the number of files. Only after having provided the robot with a sufficient number of images, the recognition tests of each subject of the group were very satisfactory.

**FIGURE 14:**
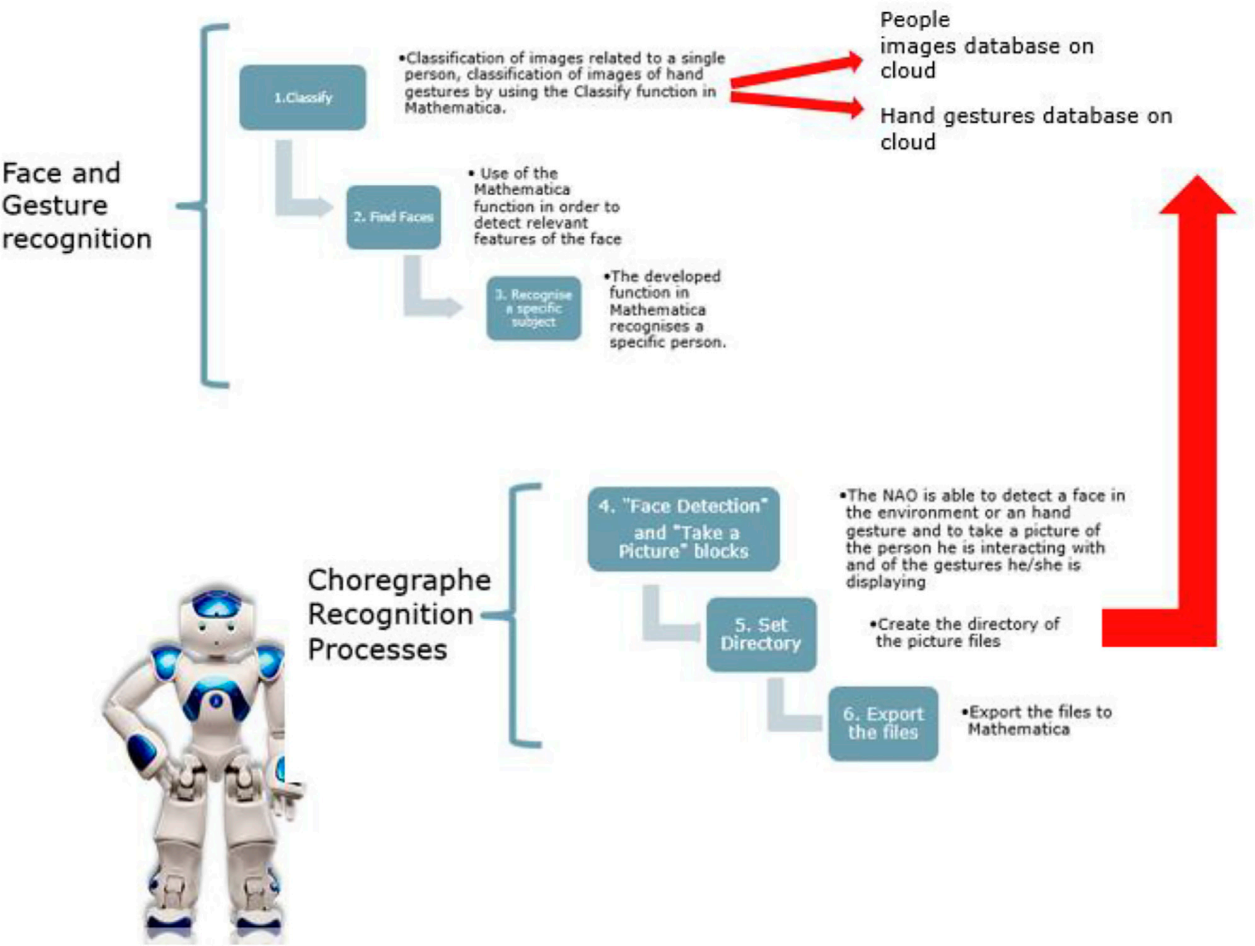
For developing the learning machine in Mathematica, a training set of photos for face recognition was created. Through the command “SetDirectory” the photos of a person of the group were imported and the face acquisition procedure was carried out with the function “FindFaces”. This procedure is repeated in the program for all classes of persons to be recognised by the robot. The last step is programming in Choregraphe. Using the “Face Detection” and “Take a Picture” blocks, the NAO searches for a face in the environment and takes a picture of she/he. These photos are saved in the NAO’s internal memory, passed to Mathematica and processed. The response file given by Mathematica is passed to Choreographe thanks to the “Get Attached” block. The file is read by “Get String”, programmed specifically in Python, and then played back through “Say Text".

The last step concerns the programming in Choregraphe; through special blocks, programmed in Python, NAO takes pictures of the faces of the people it meets and saves them on the cloud. By the communication on the server the students realized, the files are sent to Mathematica where the machine learning performs the recognition test and returns as output a text file containing the name of the recognized people that the robot will then pronounce orally. The above-discussed processes are represented in Figure 14.

From a computational skills perspective, this group of students developed a variety of machine learning to recognize both the faces of all the people in the group and hand gestures. In Mathematica, features to recognize faces and other relevant part of the human body are very effective. The main commands are related to find the coordinates of faces in an image, extracting sub images that include the detected faces, or detecting and highlighting a face in an image, returning age, sex and emotions that the faces display.

From the experimental point of view, the face recognition of the group participants was very effective. After the training performed, the machine learning recognized all 18 members of the workgroup. The subjects were recognized both in photos and in real situation, while the participants to the groups were meeting in the laboratory.

### Group E Emotion Recognition and Exhibition

Emotion recognition is already present in the NAO and is a key factor in both human and human-robot social interaction and communication.

**FIGURE 15:**
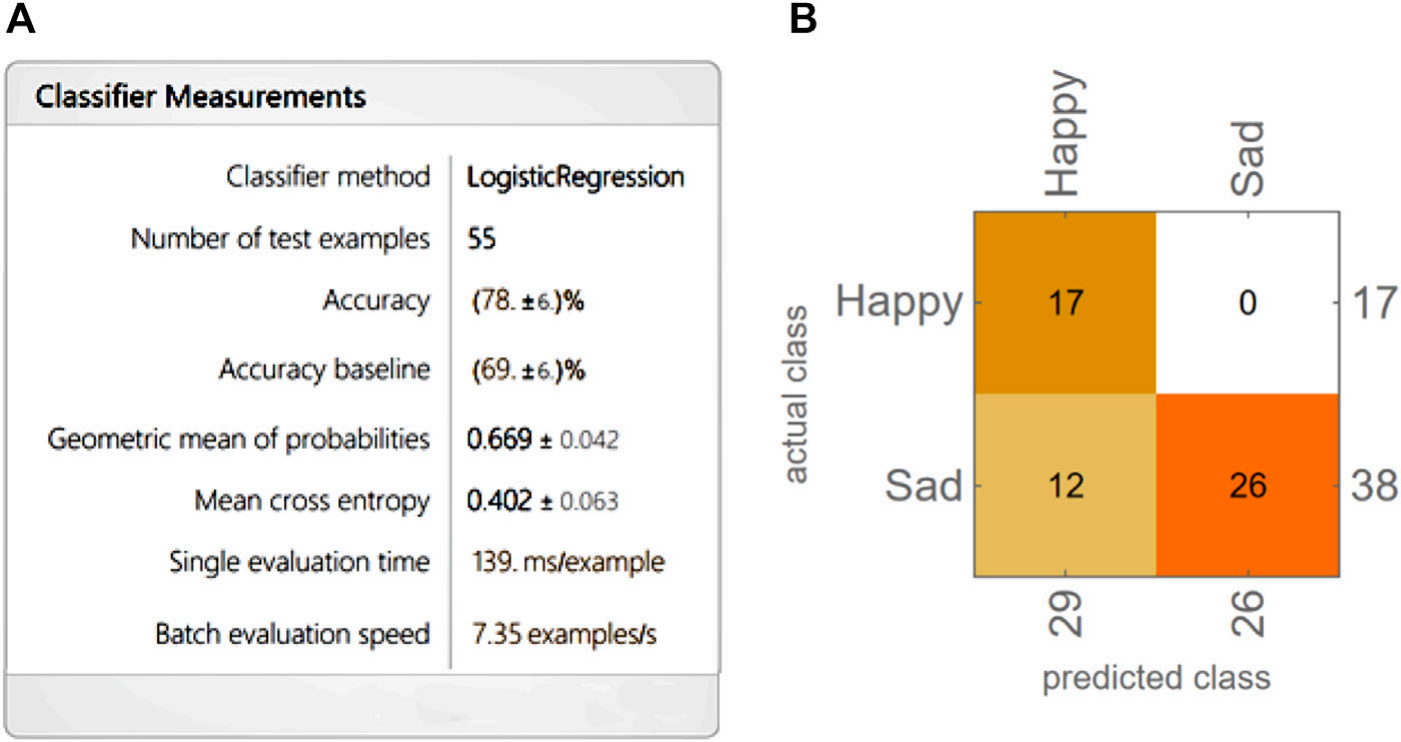
**(A)**. Classifier developed in Mathematica to analyze a test of 55 images to test recognition of the emotions Sad/ Happy. **(B)**. Confusion matrix.

To improve and optimize the performance of the robot, students in this group created an image database to test the implementation of machine learning developed in Mathematica. The database consists of 250 images of happy faces and 250 of sad faces. To test the model, a set of 55 both sad and happy images was constructed and provided to the system. The choice fell on these two emotions (Sad and Happy) precisely because the students believed that they were the most useful for the robot to understand basic social interactions and also the most detectable from the point of view of the robot-human interaction. The classifier created is shown in Figure 15.

As it is possible to see, on a number of totally 55 new Sad and Happy images, the classifier used a Logistic Regression and had a Happy and Sad emotion recognition accuracy of 78%.

**FIGURE 16:**
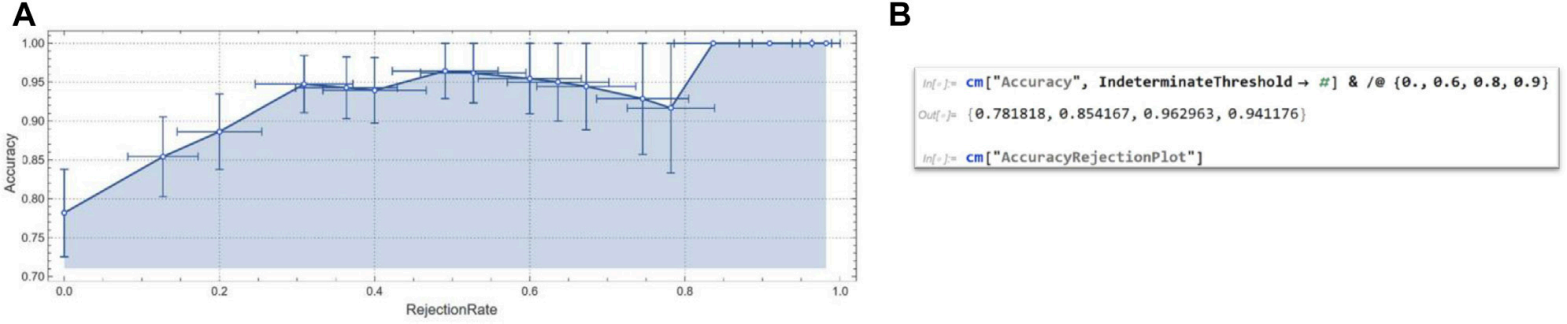
**(A)** Diagram of the Accuracy values according to RejectionRate. The graph results as the output of a Mathematica function that returns the Accuracy values and the RejectionRate for automatic sampling of the threshold values. The image from the Mathematica function is optimised according to some scientific display choices implemented by the Mathematica codes themselves and not visible to the user. **(B)** Code of the Accuracy/RejectionRate function that gives the graph reported in a.

Students also evaluated the performance of machine learning with different Indeterminacy thresholds. In Mathematica, this function, usually associated with Classify and Predict, provides an option that specifies under what probability or probability density a result should be considered indeterminate (Figure 16). In implementing the facial expression recognition system of the NAO robot, the students asked themselves: “What is the threshold of uncertainty of the system?” This question refers to the probability of recognition acceptability of the implemented algorithm.

The graph in Figure 16 crosses the recognition accuracy levels (fraction of correctly classified examples) of the system with the Rejection Rate. In fact, the ordinate shows the accuracy values, i.e. how accurate the implemented recognition machine is. The Rejection Rate (fraction of examples classified as Indeterminate) is the number of data rejected out of the total number of examples to be recognized by the robot. Mathematica provides a function that specifies under which probability level the answer of the classifier should be considered indeterminate (see Wolfram Research (2014b), IndeterminateThreshold, Wolfram Language function, https://reference.wolfram.com/language/ref/IndeterminateThreshold.html). The students, following the software guide (Wolfram Research, 2014a, ClassifierMeasurements, Wolfram Language function, https://reference.wolfram.com/language/ref/ClassifierMeasurements.html), tested the accuracy of the prediction at the same values {0.,0.6,0.8,0.9} used by the guide and reported the corresponding values that the system provided. In addition, by exploiting the potential of the algorithms, the students tested other values, shown in the graph in Figure 16, where a diagram of the Accuracy values according to RejectionRate is presented. The graph results as the output of a Mathematica function (see Figure 16B) that returns the Accuracy values and the RejectionRate for automatic sampling of the threshold values. The image from the Mathematica function is optimized according to some scientific display choices implemented by the Mathematica codes themselves and not visible to the user.

Therefore, all values from 0 to 1 were tested by the system, as can be seen in the graph, which, for graphical reasons, only shows a portion of the sampling carried out. By testing the system with various values of levels of indeterminacy, the accuracy of the system changes, because results below a certain level of accuracy are considered indeterminate.

In the performed experiments, the students set thresholds and the graph identifies the relationships between the accuracy and rejection rate values, determining the levels of indeterminacy of the system. It should be noted that these thresholds are determined by the context and the system developed.

This means that the students were able to analyze the results of the classifier probabilistically and thus provide the NAO with a highly specialized system for recognizing these emotions. This approach greatly improved their level of understanding of the emotion recognition problem.

**FIGURE 17:**
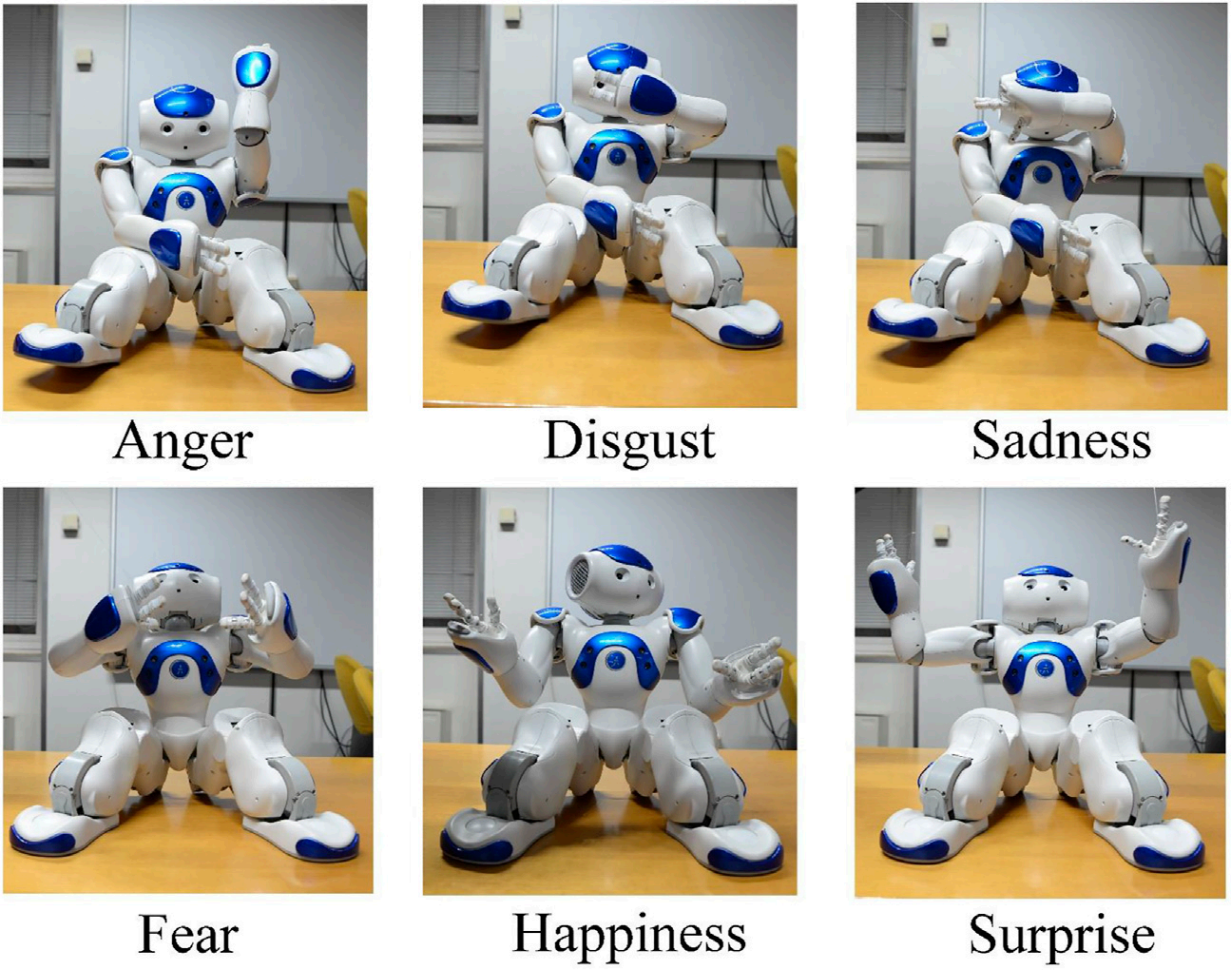
Basic six emotions displayed by the NAO Robot.

Regarding displays of emotion by the NAO in interaction with human subjects, students considered that the NAO could respond in the interaction with a series of body emotional postures that indicated, its own emotions (Erden, 2013; Saunderson and Nejat, 2019). In current literature, the emotions that the NAO can release are shown in Figure 17.

**FIGURE 18:**
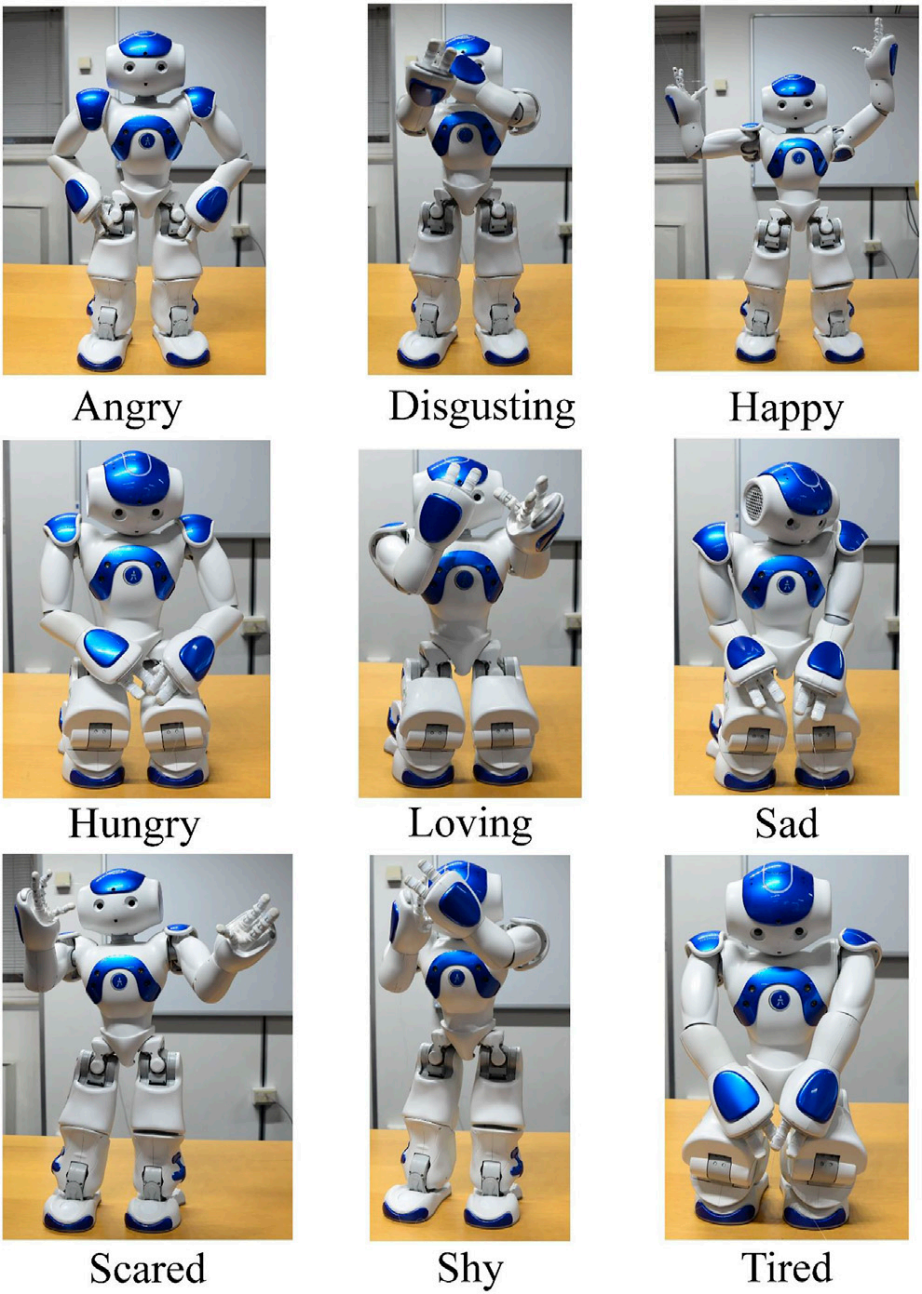
This figure reports a specification of the emotions that the NAO can express through its motor behavior.

Furthermore, the students created a program to implement a more detailed specification of the emotions the NAO can express by motor behavior, as reported in Figure 18.

**FIGURE 19:**
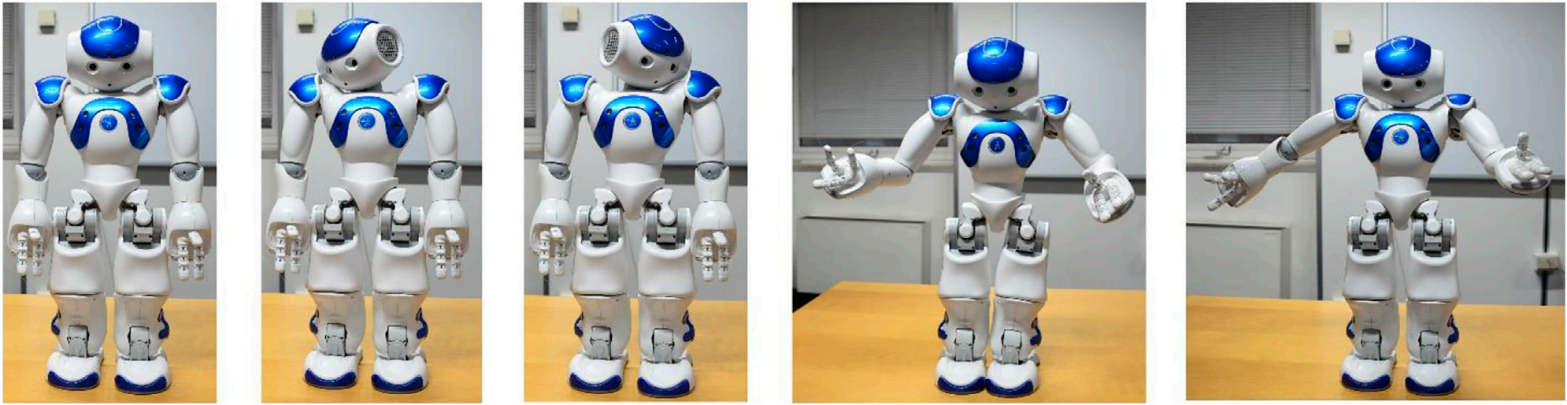
NAO emotional postures of sadness, taken from the sequential series of movements developed for this emotions.

Following Coulson (2004), the robot’s emotional postures have been implemented so that the robot moves sequentially from one to another. A video is recorded for each emotion as the robot goes through the emotional postures. In Figure 19, few frames are shown for the emotion of sadness.

The NAO robot is autonomous and communicates with Mathematica automatically. In particular, these functions are activated for behaviours that bring into play some automatic mechanisms of the system, such as recognising the presence of humans, through a series of sensors. Instead, for higher level functions, when the NAO has to control the behaviour related to motor control or social interaction, the NAO connects via its API to a set of Mathematica programs stored in a cloud. The Mathematica software processes this data and returns it to the robot. Thus, there is no interface because all functions are handled by these APIs.

**FIGURE 20:**
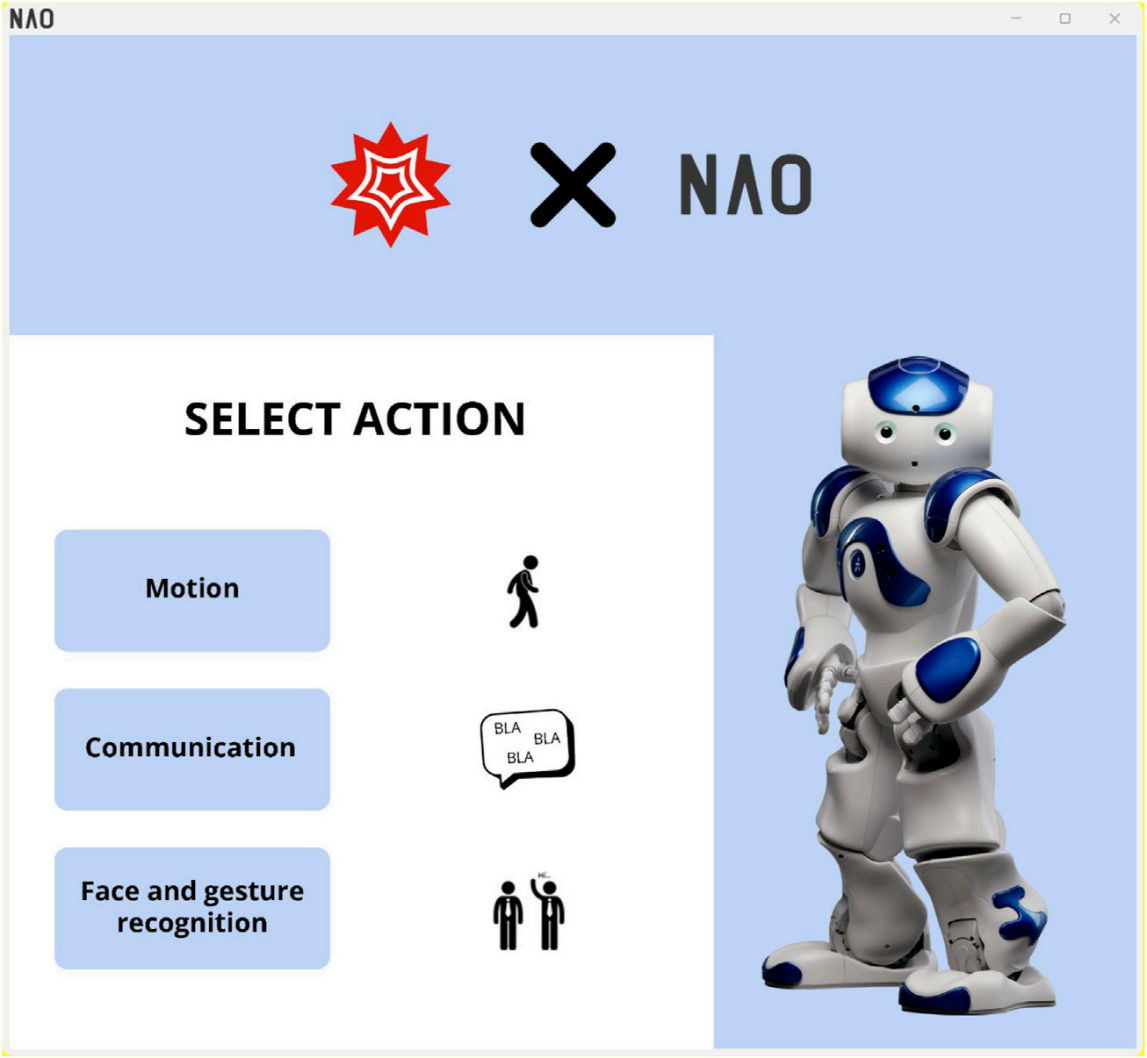
Graphical User Interface (GUI), linked to the Wolfram Mathematica software related to four NAO main functions. Each button related to the functions developed by the students’ groups are linked to the Mathematica programming environment deeply described in this paper.

APIs (which stands for Application Programming Interface) are sets of definitions and protocols with which the NAO’s application software is built and integrated. They allow the NAO to communicate with Mathematica without knowing how they are implemented, thus simplifying the processes developed in this experimentation. So, when students created the integration between the NAO and the Mathematica software, they just managed existing tools. In fact, APIs offer flexibility, simplify the design of new application, programming and use, and provide opportunities for innovation. It follows that graphical interfaces cannot be implemented. It is precisely for this reason that we have carefully described all the steps taken by the students to grasp the whole programming and control of the NAO robot. To summarise, we decided to include a figure identifying the main integration functions of the NAO applications, shown in Figure 20.

**FIGURE 21:**
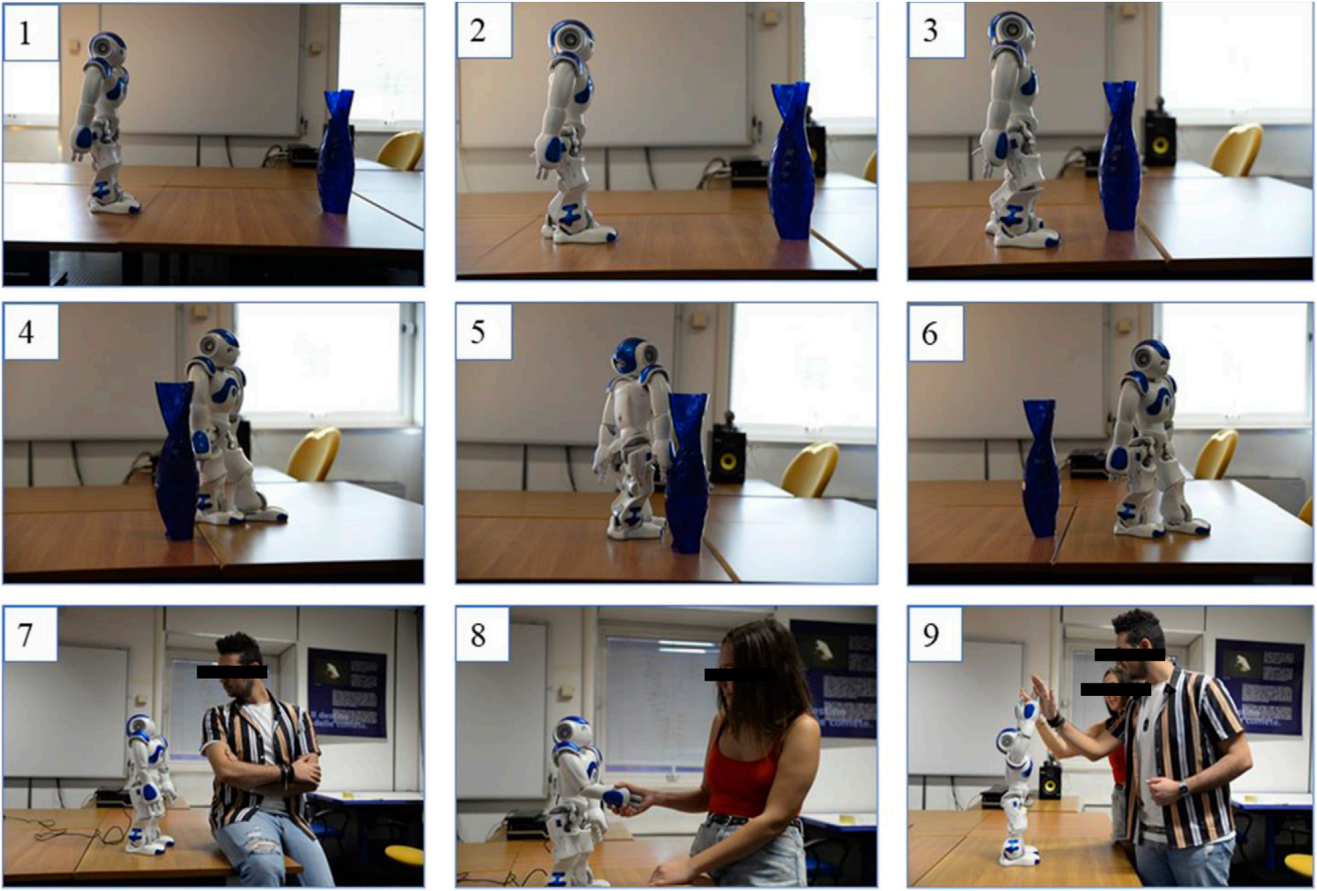
NAO’s obstacle avoiding (Steps 1–6) and social behaviour (Steps 7–9).

To exemplify the NAO social behaviour, we included a step by step collection of pictures demonstrating its main functions, reported in Figure 21.

The system is capable of interacting with the students, proceeding along trajectories and making social interaction dialogues with them. But more importantly, the development made by the various groups makes possible a very effective and fast interaction, compared to the previous programming.

## Results

Some researchers (Gabriele et al., 2017; Bertacchini et al., 2019; Gabriele et al., 2019) have highlighted that students’ computational skills are encapsulated in programs. And it is precisely the latter that are analyzed to look for whether students have achieved the goals that such educational environments propose, namely the acquisition of critical cognitive skills, which then make them solve problems. From a computational point of view, the results of this study are in line with research in the field. All students in all groups demonstrated the following computational skills, which were already partly present in their background, since they are already programmers in at least C, C++ and Python. Of these the main functions identified are as follows:

1) developing algorithms, by completing a missing step or correctly combining two algorithms; 2) evaluating algorithms, in order to fix whether an algorithm will complete the developed task or identify an inaccuracy or inefficiency in the programming, at different levels of scale; 3) developing abstractions, by describing how an algorithm or piece of code will need to be adjusted to respond to the introduction of a new variable; 4) creating documentation of implemented processes, by identifying procedures for iterative development, data collection, and documentation. From a functional point of view, we report the major functional results achieved by all groups (Table 3).

**TABLE 3:** Comparison between the basic functions of the NAO and those developed by working groups with their relative results.

Group A		
Basic function of the NAO	Function developed	Results
• Integrated programming with Python	• Creation of the communication interface between Wolfram Mathematica software and the NAO	• Improved system performance
		• Access to computational processes directly in the Mathematica cloud environment
		• Access to linguistic data resources
		• Image repositories for face and gesture recognition
		• Access to machine learning for emotion recognition
**Group B**		
Basic function of the NAO	Function developed	Results
	• Walking along complex trajectories	New trajectories
• Turn and Walk to a point		• Straight line
• Walking in circles		• Sine
• Finding the way		• Staple
**Group C**		
Basic function of the NAO	Function developed	Results
• Say anything	• Development of complex verbal dialogue	The NAO is able to answer to different questions from those that can be implemented with the basic functions in Choreographe, thus improving the social dialog in the Human-Robot Interaction
• Simple speech recognition		
• Distinguishing multiple name		
**Groups D**		
Basic function of the NAO	Function developed	Results
• Seeing face to face	• Development of faces and hand movement recognition	The NAO recognizes the faces of the 18 students belonging to the group and the hand gestures
• Seeking out face		
• Remembering face		
**Group E**		
Basic function of the NAO	Function developed	Results
• Seeing face to face	• Development of emotions recognition	Emotions recognized and mirrored
• Mirror human behavior		• Angry
		• Disgust
		• Happy
		• Hungry
		• Love
		• Sad
		• Scared
		• Shy
		• Tired

In this experiment, students not only developed computational skills, typical of educational contexts related to robotics, but also created models to describe functions of the NAO robot, describing or designing real life robotic companion. According to some researchers (Lesh et al., 2010),“A model is a system for describing (or explaining, or designing) another system(s) for some clearly specified purpose”.

The students worked building theoretical models, hypothesizing a series of working processes to connect the NAO with Mathematica. Students built models of structured knowledge, from which they made a series of logical inferences, predictions, inferred explanations for solving the problems they faced, which ultimately allowed the realization of the various steps of experimentation.

They also built empirical models, since all their working hypotheses, incorporated in the programs they developed, could be directly compared with the performance that the NAO achieved with the same programs. In fact, all of the assumptions or general principles upon which the students’ working hypotheses and then program implementation were based were tested empirically only because they incorporated an empirical model. Consequently, even the empirical data are meaningless without the interpretation provided by a model. Finally, they constructed cognitive models that were concretely embodied in the NAO robot as functions.

## The Projects Assessment

The students had to fill out reports on how they were conducting the project, explaining the choices they had made and explaining the project systematically. They made a final report in written form and a collective presentation illustrating the progress made by each group and all groups in solving the problem they were given. All notebooks developed in Mathematica program were analyzed. The performance of the robot was presented during the final exam. The students then gave a self-assessment of both the final score each deserved and the course they had attended. The list of the Assessment items is specified in Table 4.

**TABLE 4:** Assessment components of the PBL approach.

Assessment items	Details	Skills Assessed
Report	In the report, the students detailed the choice they made in order to solve the problems they encountered	Written professional communication, design skills
Notebook evaluation	Notebooks were evaluated by the 11 conceptual categories that the WL language uses	The programming expertise and the adoption of the Mathematica approach
Power point presentation	The presentation reported about all the programming and the solving of the problems	Oral communication skills
Self-assessment	Students rated how much time they had spent on the project, what the difficulty was for them, what grade they deserved	Student consistency and fairness in explaining the time and grade they deserved
Course evaluation	Students evaluated the effectiveness of the course and the contents acquired	Assessment and self-assessment skills
Robot performance evaluation	The robot performance evaluation has been done during the students presentations	The ability to cope with problems and to fulfil all the gaps the students encountered

### Students Assessment Results

As for the student **reports**, they were evaluated using the criteria of clarity and synthesis of the issues addressed. All groups wrote excellent reports, some even with experimental approaches and evaluation of robot performance (Group B). The reports also highlighted the problems encountered and the design strategies that the students implemented. The technical appropriateness of the written language and the structuring of the content was also evaluated.

**Notebooks** in Mathematica put in evidence that all groups used the conceptual categories of the Mathematica Language. They solved the problems by using complex combinations of the WL categories. The main functions used are related to the Mathematica Classify functions and the functions related to the implementation of Machine Learning systems to face the problems of automatic data recognition by the NAO robot. Very useful has been the function related to the connection with other knowledge database that Mathematica allows.

The exams were very interesting with the **presentations** given by the students. The oral communication, given the huge amount of work accomplished and the active collaboration of the students, was very well organized and clear. The presentation was very interesting and full of pictures, videos and very interesting narratives about the path followed and the collaboration needed between the groups of students to create an integrated system for the social robot. The robot performance evaluation has been done.

The students rated their effort, the time spent, the difficulty of the task very highly, and by considering all of these variables, they calculated the final grade they felt they deserved for the educational robotics lab. This **self-assessment** process usually agreed with the teacher’s evaluation. The course evaluations were also very positive. Many students found this way of learning very engaging. Being in the lab together, being able to access literature resources and read relevant literature on the problems addressed together, comparing notes with each other, being mentored by a tutor from whom to receive input and suggestions on the best design options made the lab course very interesting and motivating for all students. The evaluation of the robot’s performance was done at the same time as the presentation of the students’ projects. Therefore, the whole session was very interesting and explanatory precisely because the students gave ample and full demonstration of the improved functions of the NAO robot.

Finally, students highlighted in their reports a number of interesting aspects related to the use of WL that relate to the questions asked to understand changes (if any) in the programming approach used.

### Students Feedback on the Cognitive Use of Mathematica

A summary of the students opinion are as follows:

a. What is your opinion about the swift from the sequential programming to WL?

According to students, the transition from traditional programming languages has been difficult. New language, new grammar, new semantics to learn. Above all, what impressed them was the new conceptual framework of the program and the possibilities it provides for real-world projects. The students appreciated very much the connection with databases that WL contains and the possibility to access them with simple commands, importing symbolic objects that can already be computed in their notebooks. Another positive side, the possibility to create machine-learning systems very quickly and for the development of this function, using very few commands. Moreover, once acquired the data, it was possible for them to evaluate a series of machine learning processes, which usually in other programs require a very high programming effort. The students also appreciated the possibility to extend the functionality of the NAO in real time. Processes that usually had very high management times, resulting in inaccurate performance by the NAO robot, were speeded up, and realized in real time, thanks to their design and implementation. According to them, this really made them to fully deserve of the highest grade for the course taken.

b. What kind of cognitive processes are deployed in WL programming?

Regarding the cognitive processes used, almost all the students answered that WL allows a new way of thinking, according to them more imaginative and not based on the use of programming rules. Thanks also to the possibility of using spoken language as input, it was more necessary to create a mental model of what needed to be done, rather than thinking about the programming to be implemented. Another interesting feedback the students provided was about the ability of some functions to be easily adapted to different tasks as well. This provided a set of commands in the WL with which they could manage different processes while changing the data and functions to be performed. This process, according to them, can give rise to an internalized language, which allows them to intuitively arrive at the realization of programming problems, simply by associating the conceptual structure of functions.

c. According to your opinion, what is the role of mental representation in WL?

According to the students, the role of mental representation is very important. While in programming languages other than WL a logical sequence is required, with WL it is possible to have a block representation, and thus have a top-down view of the problem to be solved. The mental representation helped a lot in the segmentation of the problem that was given to the students. According to the students, the process of representation starting with unstructured mental images, as we progress and exchange ideas in the group, begins to grow and become more structured. At first the image seemed a bit fuzzy, but by thinking about it together and comparing notes, students shared representations and restructured the programming and functions of the robot to best solve the problem.

## Discussion and Conclusions

This experimentation involved novice student programmers, programming in the Wolfram Language (WL, Mathematica software programming language), to solve locomotion and recognition problems in real time and autonomously of the social robot NAO, thus solving real problems in an educational environment based on the Projects Based Learning method. The students, integrated in a research lab, searched for scientific articles on the field of social robotics, analysed the assigned problems, studied the NAO robot architecture and the problems of the system for a concrete use in application domains. Furthermore, as requested, they connected the robotic system to Mathematica software, and developed for the robot the skills that were necessary, namely increasing the reliability of the robot in its interaction with humans. To accomplish all these tasks, they learned to program with WL, which is completely different from the other programming languages used by the students so far.

According to the feedback and the quality of the items developed for the learning assessment provided by the PBL, students were very satisfied with the course, having learned and solved all the problems brilliantly. All of them got maximum points for the course.

While this research is still in its infancy and much work remains to be done, important conclusions can be drawn.

PBL worked and proved to be a very engaging and a motivating teaching approach for the students.

WL made students discover that they could program in a completely new way, developing critical and problem-solving skills.

## Data Availability

The original contributions presented in the study are included in the article/Supplementary Material, further inquiries can be directed to the corresponding author.
